# Toxicological and nutritional evaluation of plant cell cultures from scurvy grass (*Cochlearia danica*) and rowan (*Sorbus aucuparia*)

**DOI:** 10.3389/ftox.2025.1655489

**Published:** 2025-10-20

**Authors:** Felicianna, Ho Lam Cheung, Emily Kwun Kwan Lo, Fangfei Zhang, Hoi Kit Matthew Leung, Marsena Jasiel Ismaiah, Natalia Rosa-Sibakov, Valeria Iannone, Carlos Gómez-Gallego, Marjukka Kolehmainen, Heiko Rischer, Emilia Nordlund, Hani El-Nezami

**Affiliations:** ^1^ School of Biological Sciences, University of Hong Kong, Pokfulam, Hong Kong SAR, China; ^2^ VTT Technical Research Centre of Finland Ltd., Espoo, Finland; ^3^ Institute of Public Health and Clinical Nutrition, School of Medicine, University of Eastern Finland, Kuopio, Finland

**Keywords:** cellular agriculture, food ingredients, nutritional composition, acute toxicity, subacute toxicity, biosafety

## Abstract

**Introduction:**

Plant cell culture (PCC) technology is currently being developed to produce plant foods partially decoupled from traditional agriculture practices. By now, the safety of the ingredients produced by PCC technology for food or nutritional purposes has to be tested.

**Materials and methods:**

In this study, the oral safety and toxicity of two novel PCCs, scurvy grass (SG) (Cochlearia danica) and rowan (RW) (Sorbus aucuparia), and to characterize the macro- and micronutrient quality, including proteomic profiles, to identify potential allergens.

**Results:**

Nutritional composition analysis showed that both SG and RW PCCs profiles are comparable to other berry cell lines with a good amount of protein, dietary fibre and vitamins. Potential allergens were identified via proteomics based on structural similarity. The acute and subacute toxicity profiles of the PCC samples were evaluated based on OECD guidelines. For both PCCs, no deaths, behavioral changes, nor metabolic effects were observed at 2000 mg/kg. In the 28-day repeated oral exposure subacute toxicity study, no mortality or significant adverse clinical, hematological, or metabolic effects were observed for either SG or RW.

**Discussion:**

These findings indicate that the no-observed-adverse-effect level (NOAEL) for both PCCs exceeds 2000 mg/kg. Overall, our findings indicate that the consumption of these PCCs could be considered safe and non-toxic, although further assessments on potential allergens and phytohormone accumulation are necessary to fully ensure consumer safety. This study highlights the oral safety of PCCs for consideration as a novel food ingredient and serve as a basis for evaluating toxicological impacts of PCCs.

## 1 Introduction

The world population has increased by 14% in the past 12 years, reaching the 8.0-billion milestone in 2022, and is expected to continue, peaking at 9.7 billion in 2050 and 10.9 billion in 2100 (United Nations Department of Economic and Social Affairs, 2021). However, 9.2% of the global population was suffering from hunger in 2022 and over 3.1 billion were facing food insecurity–incapable of securing regular access to sufficient safe and nutritious food to grow, develop and lead an active and healthy lifestyle (FAO et al.,, 2023). Hence, one of the main goals of the United Nations is to achieve sustainable development by 2030, by ensuring sustainable food production systems. Currently, the increase in food production has been achieved by techniques such as breeding high-yielding crop varieties, applying inorganic fertilizers and pesticides, and by using of large-scale irrigation for monoculture farming (Shahmohamadloo et al., 2022). This approach has succeeded in improving food production, availability, and affordability, and has helped to reduce hunger and poverty to some degree. However, current agriculture practices are a major contributor to greenhouse gas emissions, habitat destruction, biodiversity loss, and soil degradation. These environmental impacts are, in turn, making agricultural landscapes increasingly vulnerable to the effects of climate change. Thus, continuing to expand global food production by bringing more land under cultivation, especially given the threats of climate change and plant diseases, is ultimately unsustainable (Mockshell and Kamanda, 2018).

Plant cell culture (PCC) technology for commercial use has been deployed since the 1980s to produce pharmaceuticals, cosmetics, and, most recently, food ingredients (Gubser et al., 2021). The advantages of PCC for food ingredient production are mainly the controlled conditions, not relying on climate, weather and soil conditions, resulting in consistent quantity and quality. The interest in PCC-based foods is emerging since the technology can enable the production of high ecological burden or endangered species (Häkkinen et al., 2020; Kobayashi et al., 2022) and provide nutritionally valuable proteins, fibres, and fatty acids (Nordlund et al., 2018; Ritala et al., 2022). PCC may also present a feasible food production system because it allows upscaling and massive production scale. For example, the production of commercialized ginseng saponins via PCC has been ongoing since the 2000s in Korea. Conventionally, the cultivation of ginseng is very labour-intensive and time-consuming–requiring a minimum of 4 years to cultivate (Wu et al., 2009). With PCCs utilizing a 500 L bioreactor, the scale up of ginseng root cell cultures to yield 149.6 g/L dry cell ginseng biomass was achieved in only 4 weeks (Choi et al., 2000). Furthermore, in PCC technology formation of beneficial substances can be promoted, while harmful substances can be limited or eliminated for the consumers. These advantages demonstrate the potential of PCCs to play a major role in attaining sustainable food production in the coming years.

Currently, two plant cell lines, scurvy grass (SG, *Cochlearia danica*, Brassicaceae) and rowan (RW, *Sorbus aucuparia*, Rosaceae), are being developed as potential novel food ingredients for commercialization at an industrial scale. These plant species are in focus due to their high protein content with a well-balanced proportion of essential amino acid content, high dietary fibre content (Ritala et al., 2022), and potential health benefits. In particular, SG is known to be vitamin C rich, while RW was demonstrated to have antidiabetic effects (Brandrud, 2014; EV et al., 2020; Wei et al., 2016; Wolf et al., 2021). Since PCC has not been used as food ingredients and are considered as novel food ingredients, the regulations require proper characterization and safety evaluation including toxicological assessment prior to commercialization (Gubser et al., 2021; Häkkinen et al., 2020). Thus, the main objectives of this study is to biochemically characterize PCC samples for the assessment of their nutritional quality; and evaluate the safety and toxicity of these mass-produced cell lines through acute and subacute oral toxicity testing.

## 2 Materials and methods

### 2.1 Production and chemical characterization of plant cell cultures

Rowan (*Sorbus aucuparia*) cell suspension culture (VTTCC P-120009) was cultivated as previously described (Ritala et al., 2022) in modified Murashige and Skoog medium (Murashige and Skoog, 1962) with 0.1 mg/L kinetin and 1 mg/L NAA. Scurvy grass (*Cochlearia Danica*, voucher specimen deposited at VTT) cell suspension culture was maintained in modified MS medium supplemented with 0.2 mg/L 2,4-dichloro-phenoxyacetic acid (Supplementary Table S1). Both cell lines were grown in 250 mL flasks on an orbital shaker (110 rpm) in darkness at 25 °C ± 1 °C and sub-culturing at 7 days intervals.

The upscaling of the studied cell lines to pilot scale was carried out in three consecutive bioreactor cultivations in working volumes of 5–6 L (Bioflo 320, Eppendorf, Germany and Biostat CT DCU3, Sartorius, Germany, respectively), 40-Dwo42 L (BioTwin42, Biostream International, Netherlands) and 200 L (Techfors, Infors HT, USA) in the same media as used for the flask cultivations. The cultivation of scurvy grass cell line was further up scaled to 1,000 L (BioFlo Pro, New Brunswick Scientific, USA). The biomass obtained from the previous cultivation was used to inoculate the following bioreactor. The cells were harvested at the early stationary phase which was determined from growth curve based on CO_2_ exhaust gas measurements. Downstream processing followed an established protocol (Nohynek et al., 2014). Shortly, biomass was separated with a Larox filter press (model PF 0.1 H2) and lyophilized (Supplementary Figure S1).

The chemical composition of SG and RW PCC samples was analyzed using standard methods for food. Briefly, moisture was determined by drying of the samples at 102** **°C–105 °C (NMKL 23:1991). The protein content was estimated by determining nitrogen content in the samples using the Kjeldahl method (NMKL 6:2003). The determination of fat content in the samples was done via acid hydrolysis followed by solvent extraction (NMKL 160:2005). For ash determination, a gravimetric method was used by ashing the sample to constant weight in a muffle furnace at 550 °C (NMKL 173:2005). The total dietary fibre was determined by simulating human digestion using enzymes to remove digestible starch and protein according to Barry et al., 2020. 25 method (Barry et al., 2020; NMKL, 1991; 2003; 2005a; 2005b). The vitamins C, D2 (ergocalciferol) and D3 (cholecalciferol), as well as E (α-, β-, γ- and δ-tocopherol) were quantified in the samples using high performance liquid chromatography according to standard method EN 12821, EN 12822 and the protocol by Fontannaz et al., 2006; CEN, 2009a, 2014; Fontannaz et al., 2006). The elemental composition of the plant cells was determined after pressure digestion by ICP-OES based on EN 15621 animal feeding stuffs, carried out by the Finnish Food Authority (CEN, 2009b).

Proteomics analysis of the PCC samples was conducted at the Proteomics Core Facility, University of Gothenburg. RW and SG lyophilized samples were homogenized using lysis matrix D (1.4 mm ceramic spheres) on a FastPrep®-24 instrument (MP Biomedicals, OH, USA). After washing, protein concentrations in the lysates were determined using Pierce™ BCA Protein Assay Kit (Thermo Fisher Scientific) and the Benchmark™ Plus microplate reader (Bio-Rad Laboratories, Hercules, CA, USA). Then, the samples were processed using modified filter-aided sample preparation (FASP) method (Wiśniewski et al., 2009). In short, samples were digested with trypsin/LysC (Promega MS grade, ratio 1:25) at 37 °C. Peptides were labelled using TMTpro 18-plex isobaric mass tagging reagents (Thermo Fisher Scientific) according to the manufacturer instructions. Peptide separation was performed using a Dionex Ultimate 3000 UPLC system (Thermo Fisher Scientific) and a reversed-phase XBridge BEH C18 column (3.5 μm, 3.0 × 150…mm, Waters Corporation) stepped gradient and Fractions were then collected and analysed on Orbitrap Lumos™ Tribrid™ mass spectrometer equipped with the FAIMS Pro ion mobility system interfaced with nLC 1200 liquid chromatography system (Thermo Fisher Scientific). Peptides were trapped on an Acclaim Pepmap 100 C18 trap column (100 μm × 2 cm, particle size 5 μm, Thermo Fisher Scientific) and separated on an in-house constructed analytical column (350 × 0.075 mm I.D.) packed with 3 μm Reprosil-Pur C18-AQ particles (Dr. Maisch, Germany) using a gradient from 3% to 80% acetonitrile in 0.2% formic acid over 90 min at a flow of 300 nL/min. Precursor ion mass spectra were acquired at 120,000 resolution, scan range 375-1,500 and maximum injection time 50 ms. The isolated precursors were fragmented by collision induced dissociation (CID) at 30% collision energy with the maximum injection time of 35 ms for 3 s (‘top speed’ setting) and detected in the ion trap, followed by multinotch (simultaneous) isolation of the top 10 MS2 fragment ions within the m/z range 400–1,400, fragmentation (MS3) by higher-energy collision dissociation (HCD) at 55% collision energy and detection in the Orbitrap at 50,000 resolution m/z range 100–500 and maximum injection time 105 ms. The data files for all fractions were merged for identification and relative quantification using Proteome Discoverer version 2.4 (Thermo Fisher Scientific). The search was against plant families Rosaceae or Brassiceae (from SwissProt, 2023-04-18), for RW and SG respectively, using Sequest as a search engine with precursor mass tolerance of 5 ppm and fragment mass tolerance of 0.6 Da. Tryptic peptides were accepted with one missed cleavage, variable modifications of methionine oxidation and fixed cysteine alkylation, TMT-label modifications of N-terminal and lysine were selected. Percolator was used for PSM validation with the strict FDR threshold of 1%.and Sequest XCorr was set on 1. TMT reporter ions were identified with 3 mmu mass tolerance in the MS3 HCD spectra, and the TMT reporter abundance values for each sample were normalized on the total peptide amount. Only the quantitative results for the unique peptide sequences with the minimum SPS match of 40% and the average S/N above 10 were taken into account for the protein quantification. The quantified proteins were filtered at 5% FDR and grouped by sharing the same sequences to minimize redundancy. The list of proteins was initially screened to include only those with a coverage greater than 10%. This criterion was established to ensure that only well-represented proteins, with a substantial portion of their sequences covered, were included in the analysis. UniProt accession numbers were then used to indicate putative allergens. Subsequently, to pinpoint probable allergens not listed in UniProt, each protein was compared with existing literature on allergens. Proteins with similarities to those documented as allergens in other organisms or species were reported. Pie charts were created by using chiplot online software (Ji et al., 2022; Li et al., 2023). The mass spectrometry proteomics data have been deposited to the ProteomeXchange Consortium via the PRIDE (Perez-Riverol et al., 2025) partner repository with the dataset identifier PXD065550.

### 2.2 Experimental animals and design

Male and female ICR-CD1 mice aged between 4–6 weeks and 25–35 g, obtained from the Centre for Comparative Medicine Research (CCMR) of the University of Hong Kong, were used in the study. The mice were acclimated for 1 week prior to experimentation. Animals were separated by sex and were housed 4-5 per cage under standard laboratory conditions with a 12-h light/dark cycle, ambient temperature of 22 °C ± 3 °C and relative humidity of 50%–60%. Drinking water and standard chow diet, comprising of 20% protein and 4.5% fat (LabDiet^®^ 5053, The Jackson Laboratory, USA) were provided *ad libitum*. After 1 week of acclimatisation, the mice were trained for voluntary oral administration as previously described^8^. In brief, mice were fasted for 16 h with drinking water provided *ad libitum* prior to the 4-day training period, in which mice were separately given plain MediGel Sucralose (ClearH_2_O, USA) daily. All protocols involved in this experiment were approved by the Committee on the Use of Live Animals in Teaching and Research (CULATR) of the University of Hong Kong (CULATR Ref. No.: 6104-22).

### 2.3 Acute oral toxicity study

**FIGURE 1:**
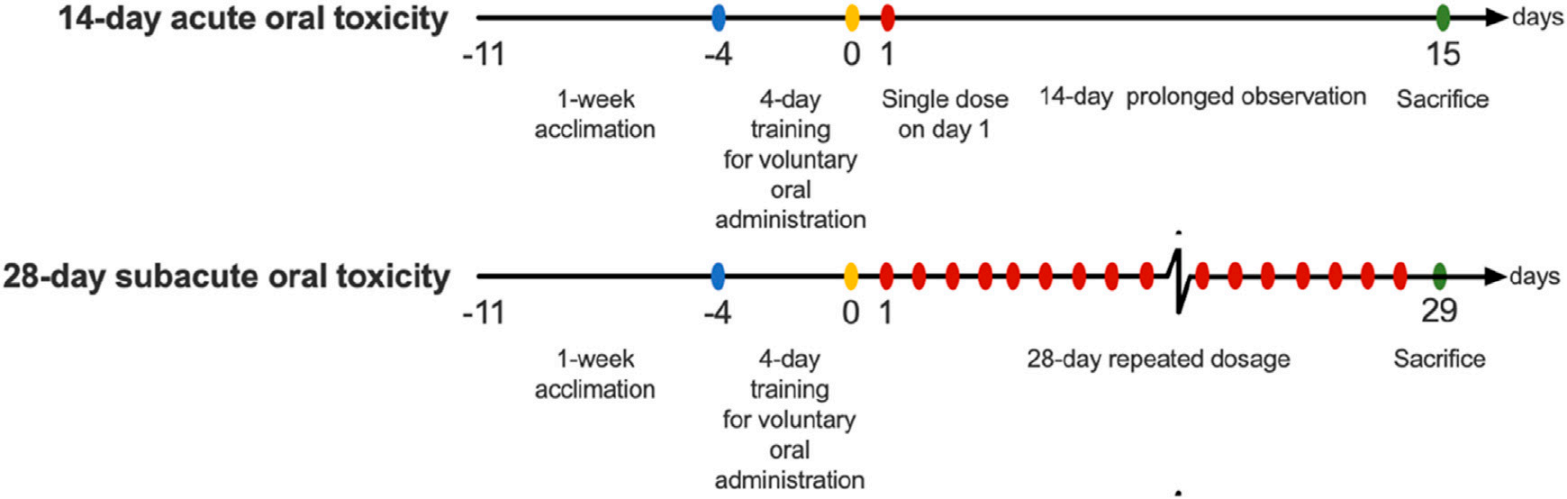
Schematic diagram of the acute and sub-acute oral toxicity studies.

Acute oral toxicity study was designed based on the guidelines provided by the Organisation for Economic Co-operation and Development No.423 (OECD 423), Acute Oral Toxicity - Acute Toxic Class Method (OECD, 2002b). Prior to the main acute oral toxicity study, a pilot sighting study was conducted–i.e., the limit test (OECD, 2002b). The limit test was adopted for the main acute toxicity study. ICR-CD1 mice were randomly assigned to 3 groups: (i) control; and the treatment groups: (ii) scurvy grass (SG) group and (iii) rowan (RW) group (*n* = 6/group). The control group was administered with the vehicle, plain MediGel, while the treatment groups with 2000 mg/kg body weight of SG or RW powder incorporated into the MediGel. A single oral administration was given, followed by a 14-day observation period (Figure 1). Body weight and surface temperature were measured daily. Weekly food consumption was measured and the mean reported. Observations of clinical toxicity and mortality signs, including changes in skin, fur, eyes, mucous membranes, occurrence of secretions and excretions and autonomic activity (e.g., lacrimation, piloerection, pupil size, unusual respiratory pattern), were monitored twice per day. Changes in gait, posture and response to handling as well as the presence of clonic or tonic movements, stereotypies (e.g., excessive grooming, repetitive circling) or bizarre behaviour (e.g., self-mutilation, walking backwards) were also monitored.

### 2.4 Subacute oral toxicity study

Subacute oral toxicity study was performed based on the guidelines provided by the Organisation for Economic Co-operation and Development No.407 (OECD 407), Repeated Dose 28-day Oral Toxicity Study in Rodents (OECD, 2008). For each study (i.e., SG and RW), male and female ICR-CD1 mice were randomly assigned to 4 groups. The first male and female group: the control group (*n* = 5–6/gender/group), was administered with vehicle MediGel via voluntary oral administration. While the rest 3 groups (*n* = 5–6/gender/group) serve as treatment groups for the administration of graded doses of either SG or RW incorporated into the MediGel at 500, 1,000 and 2000 mg/kg. Animals were dosed daily for 28 days (Figure 1). Body weight, surface temperature and food consumption were measured weekly, and animals were observed twice daily for signs of clinical toxicity and mortality.

#### 2.4.1 Haematological and biochemical parameter assessments

At the end of the experimental period, animals were euthanized with an overdose of phenobarbital via intraperitoneal injection. Blood from the inferior vena cava was collected and kept in EDTA-coated blood tubes (K_2_EDTA Capillary Collection Tube, MiniCollect^®^) for immediate haematology analysis and for the estimation of biochemical parameters. Auto haematology analyser (RT-7600S, Rayto) was utilised to measure red blood cell (RBC) and white blood cell (WBC) lymphocyte (LYM), mid cell (MID), granulocyte (GRA), platelet and haemoglobin (Hb) counts, haematocrit (Hct), mean corpuscular haemoglobin (MCH), mean corpuscular haemoglobin concentration (MCHC), mean corpuscular volume (MCV) and platelets. Subsequently, blood samples were centrifuged at 2000 g at 4 °C for 15 min for plasma isolation for the estimation of biochemical parameters and prothrombin time test. Plasma levels of total protein (TP), albumin (ALB), aspartate aminotransferase (AST), alanine aminotransferase (ALT), cholesterol (CHOL), triglycerides (TRIGL), high-density lipoproteins (HDL), low-density lipoprotein (LDL), uric acid (UA), creatinine (CREJ) and glucose (GLU) were measured using automated analyser (Cobas c111 Analyzer, Roche Diagnostics). For the measurement of plasma sodium (Na+) and potassium (K+) ion levels, plasma samples were first diluted by 10-fold. 10 μL of diluted plasma is added to 15 mL centrifuge tubes and digested in the presence of 362 µL hydrogen peroxide (H_2_O_2_) and 1,086 µL of 67%–69% concentrated nitric acid (HNO_3_) for 90 min in water bath at 60 °C. The mixture is cooled down by adding Milli Q water to make up to 15 mL. Levels of Na+ and K+ ions in the resulting solutions were measured using ICP-MS (7900 ICP-MS, Agilent). Prothrombin time test was conducted using commercially available kits (Phosphoplastin RL, r^2^ Diagnostics), and the rest of the unused plasma samples were stored at −80 °C for further analysis.

#### 2.4.2 Organ-body weight ratio and histopathological assessment

Subsequent to euthanization, vital organs, including the brain, heart, lungs, liver and kidneys, spleen, thymus and gonads were harvested and weighted. Organ-body weight ratio was determined by dividing the weight of the organ (mg) against the body weight (BW) (g) of the animal prior to sacrifice. Tissue pieces were then fixed in 10% paraformaldehyde and were processed according to standard protocols by using graded levels of ethanol and embedded in paraffin for sectioning. Approximately 4 μm sections of tissues were stained with haematoxylin and eosin for histological observation analysis. Visualization of stained sections was performed using light microscopy, and microphotographs were obtained using a Nikon DS-Ri2 camera. Visual histopathological assessments were performed according to previously established guidelines with some modifications (Liang et al., 2014; Nyska, 2023; Passmore et al., 2018; Peter and Rao, 2023; Strbe et al., 2021; Takahashi and Fukusato, 2014). In brief, for every tissue specimen, two slides were acquired for observation and grading and the mean score was obtained. For the histopathological grading of the brain, the traditional 3 cross sections approach for the grading of each brain tissue sample were performed and graded for necrosis and haemorrhage. Each heart tissue section was evaluated for lesions, necrosis and myocardium haemorrhage. For the histology scoring of lung tissues, sections of the left, right lower and upper lobe lung were graded for vascular and alveolar features, and bronchiole pathology. Meanwhile, liver tissue sections were graded for steatosis, fibrosis, lobular inflammation and ballooning, and the kidneys were graded for glomerular cell proliferation, leukocyte exudation, cellular crescents, hyaline deposits, glomerular sclerosis and tubular atrophy. Spleen and thymus were graded based on one criterion: lymphoid depletion. For the histopathological grading of gonads, the testes were evaluated for testicular damage and spermatogenesis, and the ovaries were evaluated for haemorrhage, congestion, follicular degeneration and inflammation. Specific parameters and grading used for the scoring system of each tissue can be found in Supplementary Table S2.

### 2.5 Statistical analysis

All data of the toxicity studies are expressed as means ± standard error of mean, while the biochemical composition is expressed as mean ± standard deviation (SD). Statistical analyses were all performed using GraphPad Prism 9 (GraphPad Software, LLC.). In acute oral toxicity studies, comparison of treatment mean against control mean were performed using Student’s t-test. In subacute oral toxicity studies, for each gender, comparisons of treatment means were performed against control mean, using one-way ANOVA with Šídák multiple comparisons test.

## 3 Results

### 3.1 Chemical composition and proteomic profiles of plant cell cultures

SG cells had higher protein (44.1%) and ash (7.0%) contents, which were almost double than in RW. On the other hand, RW sample had higher DF content than SG, which was mostly present in insoluble form in both plant cell samples (Table 1). SG cells had a relevant amount of vitamin C (382 mg/100 g). RW cells contained vitamins E and D3, which were absent in scurvy grass (Table 1). Concerning minerals, SG cells accumulated more calcium, iron, magnesium, molybdenum, phosphorus and potassium than RW (Table 1). None of the plant cells have accumulated heavy metals.

**TABLE 1:** Macronutrients, vitamins and minerals of plant cell cultures.

Nutrient	Rowan	Scurvy grass	DRVs* (UL)**
Protein (g/100 g)	23.9 ± 1.7	44.1 ± 3.1	
Moisture (g/100 g)	7.2 ± 0.9	4.8 ± 0.6	
Fat (g/100 g)	2.9 ± 0.3	2.0 ± 0.2	
Ash (g/100 g)	4.3 ± 0.5	7.0 ± 0.8	
Total DF *(insoluble;soluble)* (g)	27.5 *(24.0; 3.5)*	21.0 *(17.9; 3.1)*	
Vit C (ascorbic acid) (mg)	126 ± 12.6	382 ± 38.2	80 mg
Vit E (α-tocopherol) (mg)	1.04 ± 0.16	<0.08 (LOQ)	11 mg (300 mg)
Vit D2 (ergocalciferol) (µg)	<0.25 (LOQ)	<0.25 (LOQ)	15 µg (100 µg)
Vit D3 (cholecalciferol) (µg)	16.8 ± 4.3	<0.25 (LOQ)	
Elements (mg/100g)			
Calcium	300	390	860 mg (2500 mg)
Copper	<0.5	<0.5	1.3 mg (5 mg)
Iron	9	19	7 mg
Magnesium	100	150	300 mg (250 mg)
Manganese	11	17	3 mg
Molybdenum	0.49	0.84	0.065 mg (0.6 mg)
Phosphorus	280	1100	550 mg
Potassium	1800	2500	3500 mg
Selenium	<0.2	<0.2	70 µg (300 µg)
Sodium	18	13	2 g***
Zinc	9.5	7.8	6.2–10.2 mg**** (25 mg)
Arsenic	<0.05	<0.05	
Lead	<0.05	<0.05	
Cadmium	<0.01	<0.01	
Chromium	<0.05	<0.05	
Cobalt	<0.02	<0.02	
Nickel	<0.05	0.085	

*DRVs: Dietary reference values for a healthy adult population proposed by EFSA; **UL: tolerable upper intake level defined as the maximum chronic daily intake of a nutrient; ***safe and adequate intake; ****amount varying according to the levels of phytate intake.

The italic values refers to the “insoluble; soluble” values of total dietary fibres (DF).

**FIGURE 2:**
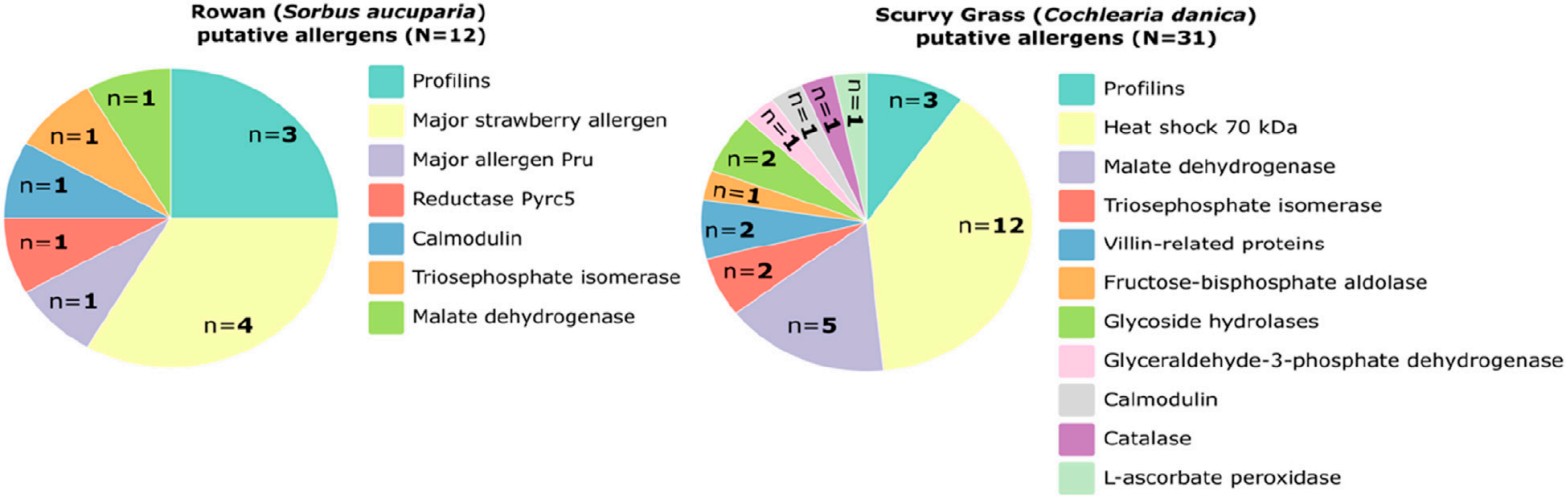
Pie chart of the proteins identified in plant cell cultures scurvy grass (*Cochlearia danica*) and rowan (*Sorbus aucuparia*) with putative allergenic potential.

In the proteomic profile of SG cells, 3991 proteins were identified and mapped in Brassicaceae family. Overall, the coverage of the protein was low, considering that 67% of the proteins showed equal or less than 10% of coverage. Among the proteins with coverage greater than 10%, three proteins belonging to the profilin family were identified, and due to their protein sequence similarity of more than 75% with the known allergen profilin-1 (Q42449, PRF1_ARATH), they may have potential allergenic properties (Figure 2; Supplementary Table S3). Additionally, 28 proteins involved in metabolism and stress response were identified as probable allergens, based on their known allergenic properties in other organisms or related plant families. In RW cells, only 63 proteins were identified and mapped to the Rosaceae family, a relatively low number that is due to the lack of a comprehensive reference genome. The protein coverage was low, with 57% of the proteins exhibiting 10% or less sequence coverage. Nevertheless, among the proteins with coverage greater than 10%, nine were classified as putative allergens (Figure 2; Supplementary Table S4), including profilins and major strawberry allergens. Furthermore, three proteins (calmodulin, triosephosphate isomerase, and malate dehydrogenase) were identified as probable allergens due to their reported allergenic potential in other species or closely related plant families.

### 3.2 Scurvy grass (SG) toxicity studies

#### 3.2.1 Acute oral toxicity

Initial pilot sighting study of the oral administration of SG at a dose of 2,000 mg/kg showed no mortality in mice. Hence, the dose: 2,000 mg/kg SG was used for the acute oral toxicity study. Administration of 2,000 mg/kg SG did not induce weight loss (Table 2), and no behavioural changes during the 14 days of observation. Body temperature, food consumption, haematology results, organs gross and histopathological analysis also showed minimal difference between the treatment and control group (Supplementary Figures S2, S3; Supplementary Tables S5, S6). Higher MCHC and relative lung weight were observed in the SG group of mice (Supplementary Tables S5,S6) but they were still within a normal range (O'Connell et al., 2015; Zhao et al., 2013). As no deaths, behavioral changes, nor metabolic effects were observed, this indicates that the median lethal dose (LD_50_) of SG exceeds 2 g/kg. According to the classification by OECD, LD_50_ being above 2 g/kg classifies this PCC into GHS Category 5 product, which signify that the product presents relatively low risk of oral acute toxicity (OECD, 2002a; 2002b).

**TABLE 2:** Effects of acute oral administration of 2000 mg/kg scurvy grass (SG) on the body weight of ICD-CD1 mice. Values are expressed as mean ± SD.

Group	Initial BW	Final BW	BW difference
Control group (n = 6)	38.72 ± 1.55	42.07 ± 2.03	+3.35
Scurvy grass (2000 mg/kg) (n = 6)	37.50 ± 0.36	40.87 ± 1.35	+3.37

#### 3.2.2 Subacute oral toxicity study

##### 3.2.2.1 Clinical observations

**FIGURE 3:**
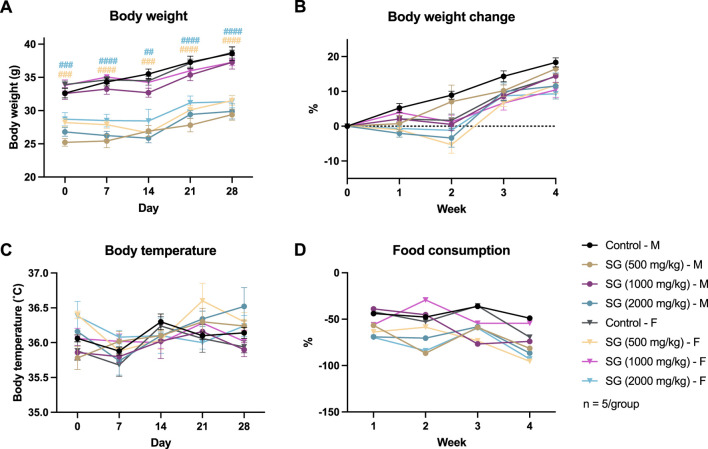
Results of the subacute oral toxicity study. **(A)** Weekly body weight. **(B)** Weekly change in body weight compared to the start of the study. **(C)** Weekly change in body temperature. **(D)** Weekly change in food consumption. *p < 0.05, **p < 0.01, ***p < 0.001, ****p < 0.0001 indicates SG administered male mice group vs. male control; ^##^ *p* < 0.01, ^###^ *p* < 0.001, ^####^ *p* < 0.0001 indicates SG administered female mice group vs. female control.

Subacute oral toxicity study on SG was performed for 28-day in a graded range of doses: 500 mg/kg, 1,000 mg/kg and 2000 mg/kg body weight. No mice died throughout the period of the study and no abnormal behavioural signs were noted prior to sacrifice. All mice exhibited body weight gain throughout the study (Figure 3A). The changes in the body weight of mice administered SG did not differ significantly with the control group, except for the male mice group administered 2000 mg/kg SG. Significant weight loss was initially observed for that group after 2 weeks of study, but the changes were within an acceptable range, i.e., ∼10%, and they recovered at week 3 and continued to gain weight until the end of study (Figures 3A,B). Meanwhile, the body temperature of mice from different groups did not vary significantly from each other, and weekly food consumption displays a large fluctuation between groups, but the trend is consistent between groups (Figures 3C,D).

##### 3.2.2.2 Haematological parameters

Haematological results are summarized in Table 3. There were no observed significant effects of SG on haematological parameters in female mice. However, in male mice, 1,000 mg/kg SG caused a significant increase in WBC, which is attributed to the increase in GRA counts (*p* < 0.001 and *p* < 0.001, respectively). Similarly, 2,000 mg/kg SG caused an increase in GRA counts but to a lesser extent than that of 1,000 mg/kg (*p* < 0.01).

**TABLE 3:** Haematological parameters of mice subjected to 28-day subacute oral toxicity test of scurvy grass (SG) at 500, 1,000, and 2000 mg/kg. *n* Values are expressed as mean ± SD.

Haematological parameter	Control	500 mg/kg	1,000 mg/kg	2000 mg/kg
Male (n = 5)				
WBCs (10^9^ g/L)	1.64 ± 0.48	1.52 ± 0.41	3.82 ± 0.93***	2.56 ± 0.90
LYM (10^9^ g/L)	0.93 ± 0.43	0.57 ± 0.11	1.74 ± 0.62	0.90 ± 0.36
MID (10^9^ g/L)	0.08 ± 0.04	0.09 ± 0.05	0.22 ± 0.09	0.14 ± 0.05
GRA (10^9^ g/L)	0.62 ± 0.17	0.85 ± 0.29	1.90 ± 0.34***	1.52 ± 0.66**
RBCs (10^12^ g/L)	7.52 ± 0.31	7.21 ± 0.72	7.66 ± 0.25	7.62 ± 0.23
HGB (g/L)	128.60 ± 3.97	126.40 ± 9.68	132.00 ± 5.83	128.00 ± 6.04
HCT (%)	42.50 ± 1.24	41.76 ± 2.68	43.58 ± 1.82	42.90 ± 1.26
MCH (pg)	17.12 ± 0.37	17.54 ± 0.64	17.22 ± 0.49	16.80 ± 0.48
MCHC (gL)	302.40 ± 6.88	302.40 ± 7.47	302.80 ± 1.10	299.00 ± 6.08
MCV (fL)	56.60 ± 2.35	58.12 ± 2.68	56.90 ± 1.66	56.26 ± 1.32
Platelets (10^9^ g/L)	793.20 ± 61.88	833.40 ± 61.07	825.00 ± 38.05	807.40 ± 57.88
Prothrombin time (s)	1.18 ± 0.04	1.07 ± 0.10	1.05 ± 0.14	1.06 ± 0.15
Female (n = 5)				
WBCs (10^9^ g/L)	2.75 ± 1.87	2.71 ± 1.31	2.19 ± 0.44	1.81 ± 0.32
LYM (10^9^ g/L)	1.62 ± 1.30	1.87 ± 1.06	1.28 ± 0.33	1.03 ± 0.27
MID (10^9^ g/L)	0.20 ± 0.22	0.11 ± 0.07	0.10 ± 0.04	0.09 ± 0.03
GRA (10^9^ g/L)	0.95 ± 0.47	0.73 ± 0.29	0.80 ± 0.28	0.69 ± 0.11
RBCs (10^12^ g/L)	7.83 ± 0.23	7.29 ± 0.30	7.54 ± 0.49	7.46 ± 0.54
HGB (g/L)	134.20 ± 3.56	128.00 ± 5.38	134.20 ± 7.97	127.00 ± 6.28
HCT (%)	44.20 ± 0.98	41.60 ± 2.03	41.66 ± 2.17	42.18 ± 2.11
MCH (pg)	17.12 ± 0.26	17.43 ± 0.57	17.62 ± 1.85	16.90 ± 0.55
MCHC (gL)	303.40 ± 8.02	305.50 ± 3.35	317.80 ± 31.475	298.00 ± 4.90
MCV (fL)	56.48 ± 1.20	57.07 ± 1.75	55.32 ± 0.84	56.68 ± 1.40
Platelets (10^9^ g/L)	778.40 ± 37.42	835.90 ± 138.51	736.40 ± 40.63	812.20 ± 46.75
Prothrombin time (s)	1.30 ± 0.03	1.22 ± 0.12	1.15 ± 0.15	1.15 ± 0.24

**p < 0.01, ***p < 0.001 vs. Control.

##### 3.2.2.3 Plasma biochemical parameters

The results of the biochemical parameters measured for the 28-day subacute toxicity study of SG is summarized in Table 4. There were no significant SG-treatment effects on the plasma biochemical parameters observed in both male and female mice.

**TABLE 4:** Biochemical parameters of ICR-CD1 mice subjected to 28-day subacute oral toxicity test of scurvy grass (SG) at 500, 1,000, and 2,000 mg/kg. Values are expressed as mean ± SD.

Biochemical parameter	Control	500 mg/kg	1,000 mg/kg	2000 mg/kg
Male (n = 5)				
Na+ (meq/L)	221.19 ± 45.43	424.53 ± 179.59	493.49 ± 328.58	179.22 ± 57.18
K+ (meq/L)	30.50 ± 6.31	43.58 ± 16.73	51.93 ± 25.17	28.87 ± 8.55
TP (g/L)	47.10 ± 3.60	47.00 ± 4.42	46.20 ± 5.14	40.90 ± 2.61
ALB(g/L)	27.37 ± 2.08	25.93 ± 3.76	26.36 ± 3.29	26.15 ± 2.53
ALT (U/L)	36.10 ± 23.39	60.60 ± 31.70	39.90 ± 9.24	48.50 ± 24.86
AST (U/L)	56.60 ± 16.20	77.60 ± 27.15	62.60 ± 19.74	61.30 ± 20.99
CHOL (mmol/L)	2.47 ± 0.54	2.30 ± 0.30	1.62 ± 1.38	2.56 ± 0.90
TRIGL (mmol/L)	1.19 ± 0.38	1.43 ± 0.69	1.11 ± 0.77	1.09 ± 0.19
HDL (mmol/L)	1.89 ± 0.35	1.84 ± 0.29	1.93 ± 0.39	1.83 ± 0.82
LDL (mmol/L)	0.22 ± 0.13	0.15 ± 0.05	0.20 ± 0.14	0.21 ± 0.12
CREJ (µmol/L)	17.10 ± 5.80	18.42 ± 1.89	14.62 ± 1.19	18.84 ± 3.97
GLU (mmol/L)	15.22 ± 3.16	13.54 ± 2.39	11.86 ± 2.85	14.23 ± 2.21
UA (µmol/L)	97.40 ± 34.14	109.40 ± 50.20	89.90 ± 56.35	85.50 ± 34.24
Female (n = 5)				
Na+ (meq/L)	193.74 ± 26.19	237.73 ± 86.4	218.15 ± 35.61	195.80 ± 42.00
K+ (meq/L)	39.30 ± 8.71	37.20 ± 15.47	35.14 ± 17.15	36.89 ± 11.35
TP (g/L)	50.00 ± 3.81	52.40 ± 1.52	60.70 ± 32.46	49.10 ± 3.80
ALB(g/L)	30.10 ± 6.56	30.76 ± 1.05	29.73 ± 1.38	31.00 ± 5.62
ALT (U/L)	32.90 ± 9.43	41.60 ± 18.67	22.30 ± 2.61	29.20 ± 5.94
AST (U/L)	65.60 ± 11.33	77.80 ± 24.81	42.70 ± 5.14	56.10 ± 7.88
CHOL (mmol/L)	2.20 ± 0.58	2.01 ± 0.16	1.85 ± 0.40	2.04 ± 0.52
TRIGL (mmol/L)	0.94 ± 0.16	1.09 ± 0.52	0.95 ± 0.29	0.56 ± 0.16
HDL (mmol/L)	1.40 ± 0.13	1.36 ± 0.15	1.10 ± 0.18	1.46 ± 0.48
LDL (mmol/L)	0.27 ± 0.08	0.21 ± 0.07	0.24 ± 0.10	0.31 ± 0.08
CREJ (µmol/L)	16.84 ± 2.62	16.94 ± 7.43	44.64 ± 66.34	25.46 ± 6.66
GLU (mmol/L)	15.05 ± 3.23	16.49 ± 3.18	12.97 ± 3.01	13.32 ± 2.23
UA (µmol/L)	124.10 ± 47.54	131.50 ± 77.69	116.90 ± 61.05	91.20 ± 54.79

No statistical significance noted between the control group and the treatment group.

##### 3.2.2.4 Organ-body weight ratio and histopathological analysis

**FIGURE 4:**
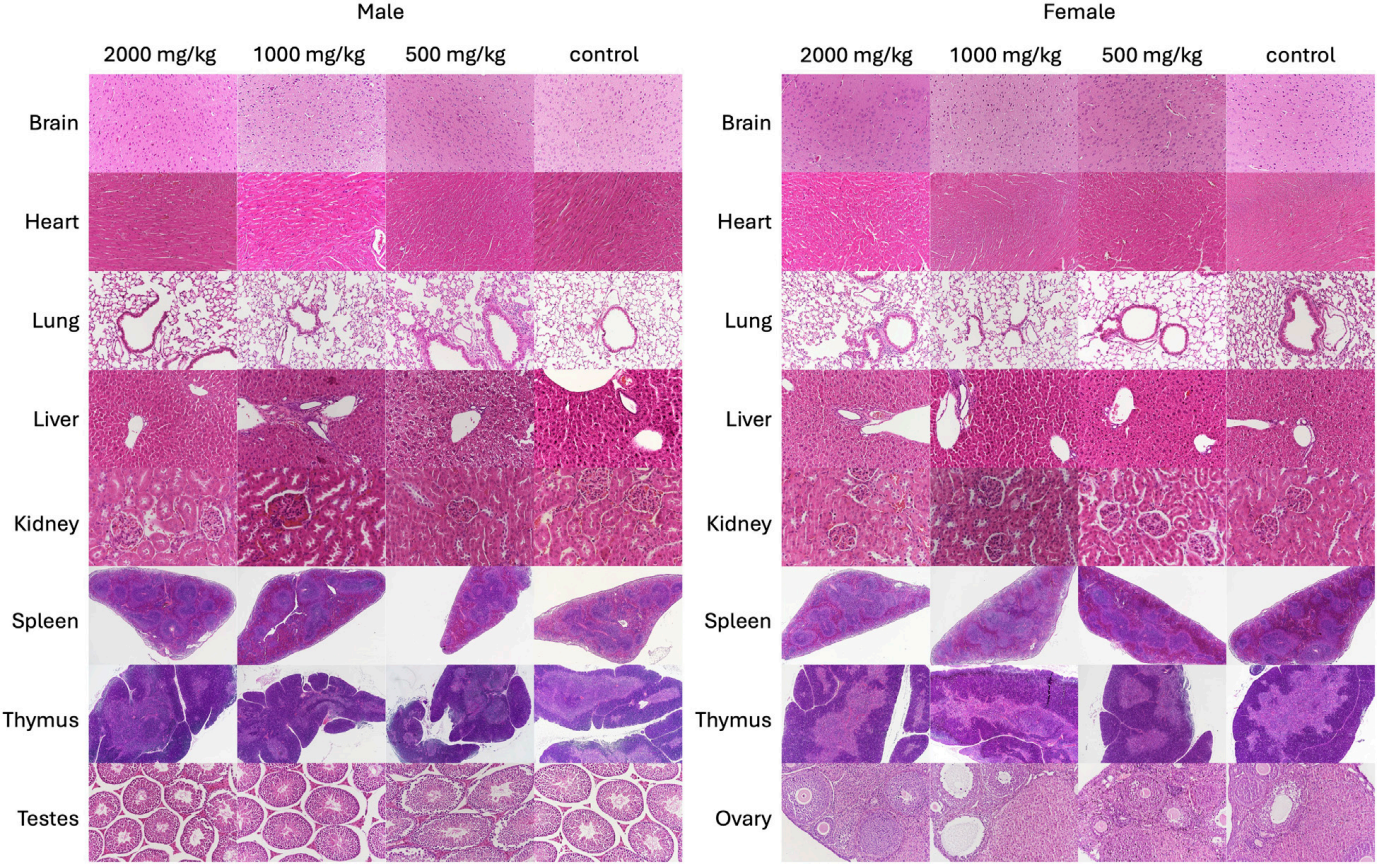
Representative haematoxylin and eosin-stained sections of brain (2000×), heart (2000×), lungs (2000×), liver (2000×), kidneys (2000×), spleen (400×), thymus (400×) and testes (2000×) of control and SG-treated mice subjected to the 28-day subacute oral toxicity study of scurvy grass (SG).

Relative organ weights between groups showed a marginal difference. In male mice, relative lung weights were increased significantly (*p* < 0.01) in the 2,000 mg/kg SG administered mice, and relative liver weights were increased significantly (*p* < 0.05) in the 1,000 mg/kg and 2000 mg/kg SG administered mice. In female mice, no significant differences were noted between the relative weight of organs except the significant relative enlargement of ovaries (*p* < 0.05) in mice administered 1,000 mg/kg SG (Table 5). These findings indicate a potential toxic effect of SG on the liver and lungs. Nonetheless, gross pathology assessments of organs showed no significant effects on the administration of SG (Figure 4; Supplementary Figure S4).

**TABLE 5:** Organ-body weight ratio of ICR-CD1 mice subjected to 28-day subacute oral toxicity study of scurvy grass (SG) at 500, 1,000, and 2,000 mg/kg vs. control. Values are expressed as mean ± SD.

Organ-body weight ratio	Control	500 mg/kg	1,000 mg/kg	2000 mg/kg
Male (n = 5)				
Brain	1.21 ± 0.08	1.29 ± 0.09	1.23 ± 0.12	1.33 ± 0.15
Heart	0.53 ± 0.03	0.51 ± 0.05	0.51 ± 0.09	0.48 ± 0.06
Lungs	0.59 ± 0.04	0.69 ± 0.07	0.66 ± 0.08	0.74 ± 0.06
Liver	5.45 ± 0.28	6.12 ± 0.28	6.29 ± 0.60	6.22 ± 0.46
Kidneys	1.56 ± 0.14	1.59 ± 0.10	1.64 ± 0.08	1.70 ± 0.08
Spleen	0.30 ± 0.04	0.28 ± 0.04	0.26 ± 0.05	0.29 ± 0.04
Thymus	0.10 ± 0.03	0.12 ± 0.04	0.11 ± 0.05	0.11 ± 0.03
Testes	0.73 ± 0.10	0.78 ± 0.09	0.89 ± 0.13	0.75 ± 0.04
Female (n = 5)				
Brain	1.62 ± 0.09	1.67 ± 0.11	1.49 ± 0.07	1.63 ± 0.11
Heart	0.53 ± 0.09	0.49 ± 0.07	0.45 ± 0.02	0.53 ± 0.09
Lungs	0.81 ± 0.07	0.76 ± 0.07	0.72 ± 0.06	0.85 ± 0.03
Liver	5.26 ± 0.28	5.90 ± 0.38	5.48 ± 0.49	5.35 ± 0.73
Kidneys	1.26 ± 0.13	1.35 ± 0.09	1.25 ± 0.12	1.38 ± 0.22
Spleen	0.38 ± 0.08	0.42 ± 0.09	0.35 ± 0.02	0.43 ± 0.14
Thymus	0.20 ± 0.07	0.22 ± 0.09	0.14 ± 0.05	0.22 ± 0.04
Ovaries	0.06 ± 0.04	0.09 ± 0.02	0.11 ± 0.03*	0.10 ± 0.02

*p < 0.05.

### 3.3 Rowan (RW) toxicity studies

#### 3.3.1 Acute oral toxicity study

Similar to the SG acute oral toxicity study, the pilot sighting study investigating the oral administration of RW at a dose of 2000 mg/kg revealed no mortality among the treated mice. Consequently, this dose was selected for the acute oral toxicity study. The administration of 2000 mg/kg RW did not result in weight loss (Table 6), nor did it cause any behavioural changes. Additionally, there were no significant differences observed between the treatment and control groups in terms of body temperature, food consumption, haematology results, and both gross and histopathological analyses of organs (Supplementary Tables S7, S8; Supplementary Figures S5, S6, S7). Overall, these results indicate that the RW PCC falls under GHS Category 5 product, which presents a very low risk of acute toxicity (OECD, 2002a; 2002b).

**TABLE 6:** Effects of acute oral administration of 2,000 mg/kg rowan (RW) on the body weight of ICD-CD1 male mice. Values are expressed as mean ± SD.

Group	Initial BW	Final BW	BW difference
Control group (n = 6)	38.42 ± 2.98	41.43 ± 1.66	+3.02
RW (2000 mg/kg) (n = 6)	37.58 ± 3.94	40.88 ± 4.71	+3.30

#### 3.3.2 Subacute oral toxicity study

##### 3.3.2.1 Clinical observations

**FIGURE 5:**
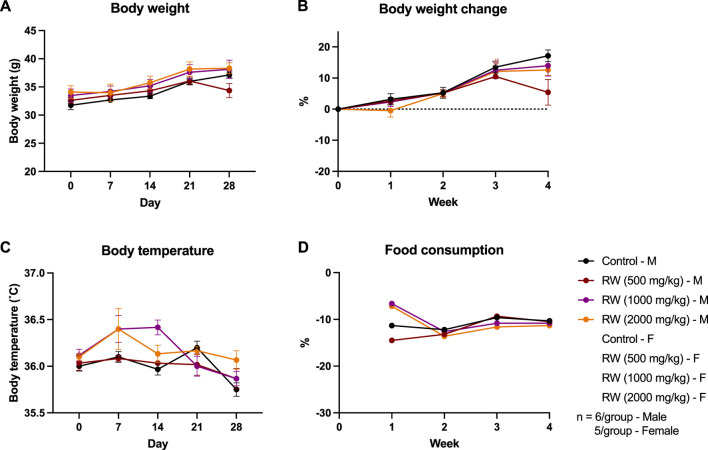
Results of the subacute oral toxicity study on RW. **(A)** Weekly body weight. **(B)** Weekly change in body weight compared to initial weight of study. **(C)** Weekly change in body temperature. **(D)** Weekly change in food consumption. *p < 0.05, ***p < 0.001 indicates RW administered male mice group vs. male control.

The 28-day subacute oral toxicity study on RW was performed in graded range of doses of 500, 1,000 and 2,000 mg/kg body weight. No mortality and abnormal behavioural signs were noted prior to sacrifice. All mice showed weight gain by the end of the study (Figures 5A,B). The variance in weight gain among different groups within each gender was generally not significant, except for female mice treated with 500 mg/kg RW, which exhibited a significant increase in body weight gain (*p* < 0.05) at week 3 compared to female control mice. Additionally, male mice treated with 500 mg/kg RW experienced a sudden weight loss (*p* < 0.05) of 10% at the final week of the subacute oral toxicity study (Figure 5B). Moreover, a significant higher body temperature was recorded in male mice administered 1,000 mg/kg RW at week 3 of the study but the measured body temperature of these mice still falls within a normal body temperature range, i.e., 36 °C–37 °C (Figure 5C), while no significant differences between weekly food consumption were noted (Figure 5D).

##### 3.3.2.2 Haematological parameters

The haematological parameters of mice subjected to a 28-day subacute oral toxicity study of RW are summarized in Table 7. No significant effects of RW were observed in the blood composition between mice administered with RW at different doses and the control mice group for both genders.

**TABLE 7:** Haematological parameters of mice subjected to 28-day subacute oral toxicity test of rowan (RW) at 500, 1,000, and 2000 mg/kg. Values are expressed as mean ± SD.

Haematological parameter	Control	500 mg/kg	1,000 mg/kg	2,000 mg/kg
Male (n = 6)				
WBCs (10^9^ g/L)	2.36 ± 0.26	1.81 ± 0.40	1.89 ± 1.33	3.20 ± 1.15
LYM (10^9^ g/L)	1.08 ± 0.25	0.85 ± 0.15	0.97 ± 0.55	1.15 ± 0.63
MID (10^9^ g/L)	0.14 ± 0.06	0.09 ± 0.03	0.14 ± 0.08	0.13 ± 0.03
GRA (10^9^ g/L)	1.14 ± 0.30	0.87 ± 0.28	1.80 ± 1.52	1.93 ± 0.63
RBCs (10^12^ g/L)	7.55 ± 0.10	7.76 ± 0.60	7.14 ± 0.82	7.19 ± 0.44
HGB (g/L)	132.50 ± 6.02	138.00 ± 12.33	123.00 ± 12.81	122.08 ± 9.09
HCT (%)	42.50 ± 1.55	43.38 ± 3.33	40.51 ± 4.41	41.16 ± 3.78
MCH (pg)	17.48 ± 0.67	17.75 ± 0.87	17.08 ± 0.86	16.93 ± 0.37
MCHC (gL)	311.00 ± 6.48	318.00 ± 13.10	303.42 ± 3.85	296.75 ± 6.80
MCV (fL)	56.27 ± 1.48	55.85 ± 1.36	56.84 ± 1.25	57.20 ± 2.32
Platelets (10^9^ g/L)	944.17 ± 93.68	956.50 ± 113.86	886.00 ± 142.28	835.17 ± 132.89
Prothrombin time (s)	0.75 ± 0.05	0.81 ± 0.06	0.77 ± 0.04	0.75 ± 0.04
Female (n = 5)				
WBCs (10^9^ g/L)	1.53 ± 0.68	1.48 ± 0.16	1.65 ± 1.17	1.49 ± 0.21
LYM (10^9^ g/L)	1.32 ± 0.88	1.05 ± 0.43	0.79 ± 0.49	0.73 ± 0.21
MID (10^9^ g/L)	0.53 ± 0.66	0.29 ± 0.25	0.10 ± 0.03	0.08 ± 0.04
GRA (10^9^ g/L)	9.36 ± 0.23	0.75 ± 0.22	0.78 ± 0.66	0.68 ± 0.18
RBCs (10^12^ g/L)	7.49 ± 0.33	7.37 ± 0.80	7.78 ± 0.58	7.86 ± 0.16
HGB (g/L)	129.10 ± 6.73	118.75 ± 21.96	130.10 ± 13.07	123.70 ± 16.01
HCT (%)	41.62 ± 1.06	40.98 ± 4.44	43.16 ± 3.47	43.48 ± 0.93
MCH (pg)	17.17 ± 0.75	16.21 ± 2.97	16,72 ± 0.55	15.70 ± 1.86
MCHC (gL)	309.60 ± 10.97	291.50 ± 50.69	301.30 ± 6.52	283.70 ± 32.36
MCV (fL)	55.49 ± 1.94	55.54 ± 0.79	55.44 ± 0.83	55.30 ± 1.05
Platelets (10^9^ g/L)	806.60 ± 53.05	800.25 ± 94.07	846.40 ± 107.50	836.60 ± 300.96
Prothrombin time (s)	0.79 ± 0.04	0.85 ± 0.07	0.86 ± 0.06	0.84 ± 0.04

No statistical significance was noted between the control group and the treatment group.

##### 3.3.2.3 Plasma biochemical parameters

The results of the biochemical parameters measured for the 28-day subacute toxicity study of RW are summarized in Table 8. Most plasma biochemical parameters (TP, ALB, ALT, AST, CHOL, TRIGL, HDL, LDL, and CREJ) did not differ significantly between groups of mice administered RW and control mice group in each gender. Sodium (Na+) levels were seen to be elevated in male mice administered with 2000 mg/kg (*p* < 0.01) and in female mice treated with 1,000 mg/kg RW (*p* < 0.0001). Potassium (K+) levels were also found to be significantly higher in female mice treated with 1,000 mg/kg RW (p < 0.01). Meanwhile, uric acid (UA) levels were found to be decreased in male mice treated with 1,000 mg/kg RW (p < 0.05), and albumin (ALB) levels were found to be decreased in female mice treated with 500 mg/kg RW (p < 0.05). However, since these effects of RW are not dose-dependent, these observed changes could be coincidental. Furthermore, all mice showed a lowered glucose level (GLU) with RW administration, and this effect was more prominent in male mice in which all three different doses of RW led to significantly decreased glucose levels (*p <* 0.01 for both 500 mg/kg and 1,000 mg/kg RW; and *p <* 0.05 for 2000 mg/kg RW), while in female mice, only 1,000 mg/kg RW significantly reduced glucose levels (*p* < 0.05).

**TABLE 8:** Biochemical parameters of mice subjected to 28-day subacute oral toxicity test of rowan (RW) at 500, 1,000, and 2,000 mg/kg. Values are expressed as mean ± SD.

Biochemical parameter	Control	500 mg/kg	1,000 mg/kg	2000 mg/kg
Male (n = 6)				
Na+ (meq/L)	40.34 ± 18.93	34.10 ± 9.81	47.48 ± 8.24	68.79 ± 10.93**
K+ (meq/L)	3.51 ± 2.15	3.48 ± 1.74	4.01 ± 0.99	5.17 ± 1.14
TP (g/L)	24.00 ± 1.34	19.08 ± 2.44	21.58 ± 1.50	19.00 ± 1.18
ALB(g/L)	29.84 ± 1.07	26.21 ± 3.84	27.94 ± 3.09	24.73 ± 1.62
ALT (U/L)	47.25 ± 28.72	36.50 ± 25.07	70.25 ± 47.33	77.33 ± 34.40
AST (U/L)	67.83 ± 11.47	97.33 ± 51.69	89.42 ± 57.44	86.25 ± 20.23
CHOL (mmol/L)	3.13 ± 0.61	2.41 ± 1.17	2.25 ± 0.81	2.55 ± 0.54
TRIGL (mmol/L)	1.45 ± 0.36	0.94 ± 0.51	1.65 ± 1.03	1.52 ± 0.48
HDL (mmol/L)	2.55 ± 0.52	2.26 ± 0.43	1.74 ± 0.80	1.96 ± 0.38
LDL (mmol/L)	0.37 ± 0.05	0.29 ± 0.10	0.27 ± 0.12	0.38 ± 0.20
CREJ (µmol/L)	18.60 ± 7.75	15.28 ± 6.66	13.02 ± 3.79	17.15 ± 6.88
GLU (mmol/L)	21.38 ± 1.26	10.30 ± 9.88**	10.10 ± 4.20**	13.00 ± 1.60*
UA (µmol/L)	167.58 ± 68.98	127.42 ± 71.34	64.83 ± 25.05*	103.75 ± 44.45
Female (n = 5)				
Na+ (meq/L)	29.31 ± 2.34	36.04 ± 9.17	95.14 ± 33.50****	58.61 ± 5.69
K+ (meq/L)	2.27 ± 0.29	3.17 ± 1.40	9.46 ± 4.70**	5.11 ± 1.67
TP (g/L)	24.30 ± 1.82	20.20 ± 3.55	23.20 ± 1.44	33.30 ± 17.29
ALB(g/L)	34.73 ± 2.81	26.15 ± 7.25*	34.70 ± 2.31	32.02 ± 1.81
ALT (U/L)	28.20 ± 11.16	16.40 ± 2.54	30.50 ± 11.58	34.10 ± 7.54
AST (U/L)	63.50 ± 21.75	41.60 ± 24.51	79.50 ± 17.23	73.60 ± 7.00
CHOL (mmol/L)	1.59 ± 1.00	1.68 ± 0.29	2.08 ± 0.97	2.02 ± 0.18
TRIGL (mmol/L)	0.68 ± 0.22	0.59 ± 0.22	0.86 ± 0.33	0.67 ± 0.19
HDL (mmol/L)	1.54 ± 0.39	1.26 ± 0.21	1.93 ± 0.41	1.57 ± 0.10
LDL (mmol/L)	1.29 ± 0.11	0.29 ± 0.12	0.26 ± 0.20	0.38 ± 0.14
CREJ (µmol/L)	77.22 ± 73.35	38.18 ± 57.37	11.74 ± 3.18	20.18 ± 8.63
GLU (mmol/L)	15.31 ± 3.89	9.45 ± 2.88	8.02 ± 6.17*	12.28 ± 2.29
UA (µmol/L)	120.70 ± 64.99	77.70 ± 44.06	99.80 ± 33.96	106.20 ± 35.61

*p < 0.05, **p < 0.01, ****p < 0.0001 vs. control.

##### 3.3.2.4 Body-organ weight ratio and histological assessment

**FIGURE 6:**
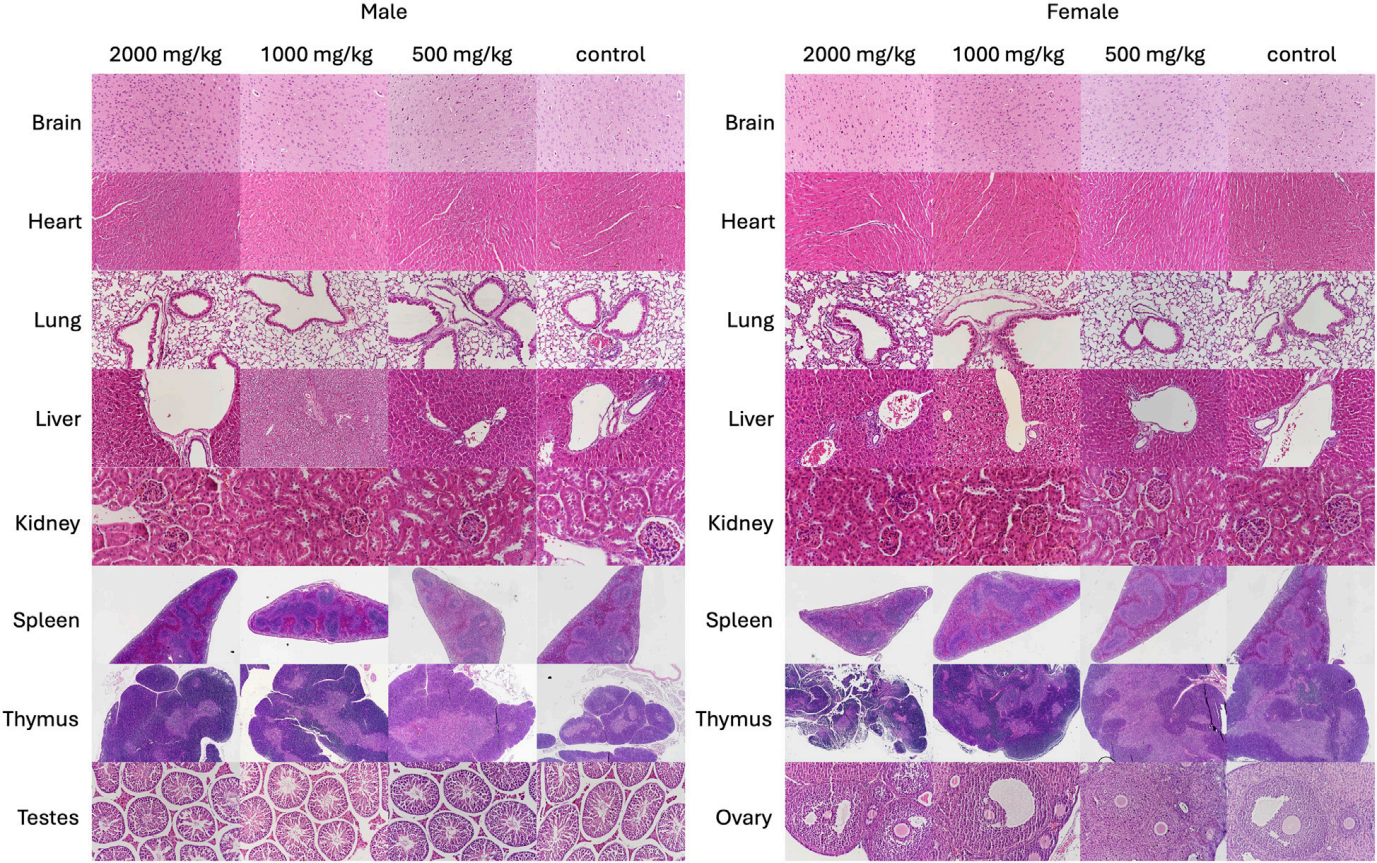
Representative haematoxylin and eosin-stained sections of brain (2000×), heart (2000×), lungs (2000×), liver (2000×), kidneys (2000×), spleen (400×), thymus (400×) and testes (2000×) of control and rowan (RW)-treated mice in the 28-day subacute oral toxicity study.

Relative organ weights of mice subjected to the 28-day subacute oral toxicity study of RW is summarized in Table 9. There were no differences between the relative organ weights between RW-treated groups and the control groups for both mice genders. Additionally, there were no significant effects observed on gross pathology assessments of organs of mice administered RW (Figure 6; Supplementary Figure S5). Overall, these findings indicate that RW oral administration likely does not produce any significant toxicity in these major organs.

**TABLE 9:** Organ-body weight ratio of ICR-CD1 mice subjected to 28-day subacute oral toxicity study of rowan (RW) at 500, 1,000, and 2,000 mg/kg vs. control. Values are expressed as mean ± SD.

Organ‐body weight ratio	Control	500 mg/kg	1,000 mg/kg	2,000 mg/kg
Male (n = 6)				
Brain	1.29 ± 0.14	1.34 ± 0.11	1.34 ± 0.20	1.27 ± 0.06
Heart	0.47 ± 0.05	0.48 ± 0.08	0.45 ± 0.04	0.48 ± 0.04
Lungs	0.64 ± 0.06	0.71 ± 0.06	0.63 ± 0.06	0.65 ± 0.04
Liver	6.14 ± 0.25	5.42 ± 0.93	6.04 ± 0.74	5.62 ± 0.85
Kidneys	1.56 ± 0.21	1.70 ± 0.21	1.60 ± 0.04	1.55 ± 0.11
Spleen	0.33 ± 0.06	0.27 ± 0.10	0.32 ± 0.20	0.26 ± 0.10
Thymus	0.21 ± 0.05	0.08 ± 0.02	0.12 ± 0.04	0.07 ± 0.02
Testes	0.80 ± 0.10	0.90 ± 0.13	0.80 ± 0.17	0.80 ± 0.09
Female (n = 5)				
Brain	1.60 ± 0.11	1.54 ± 0.29	1.60 ± 0.10	1.53 ± 0.08
Heart	0.46 ± 0.03	0.49 ± 0.04	0.51 ± 0.01	0.49 ± 0.04
Lungs	0.62 ± 0.31	0.54 ± 0.42	0.56 ± 0.28	0.59 ± 0.29
Liver	4.70 ± 0.18	4.59 ± 0.25	4.44 ± 0.27	4.83 ± 0.50
Kidneys	1.14 ± 0.08	1.20 ± 0.12	1.22 ± 0.12	1.18 ± 0.12
Spleen	0.42 ± 0.05	0.35 ± 0.01	0.30 ± 0.08	0.25 ± 0.07
Thymus	0.15 ± 0.10	0.11 ± 0.06	0.16 ± 0.10	0.11 ± 0.06
Ovaries	0.27 ± 0.36	0.25 ± 0.36	0.28 ± 0.37	0.22 ± 0.29

No statistical significance noted between the control group and treatment group.

## 4 Discussion

PCC technology is a new food production method that can contribute to the development of sustainable food production systems globally. The PCC samples investigated in this work have potential as novel food ingredients due to their considerable amount of relevant macro- and micronutrients. However, as they are considered novel foods by the regulation, proper characterization and safety evaluation are required to enable the commercialization of the ingredients for food use. Specifically, according to article 7 in the EU legislation on novel foods, PCCs could only be authorized when scientific evidence shows that they do not pose a safety risk to human health, and in case its intended use is to replace another food, its nutrition value should not differ significantly, and that its intended normal consumption should not be nutritionally disadvantageous compared to the original food (Regulation 2015/2283) (European Union, 2021). Therefore, this study focused on increasing the understanding of the nutritional and safety quality of the PCC samples.

The substantial amount of DF in RW cells was confirmed from a previous study (Ritala et al., 2022), and DF in SG cells was in a similar range as previously reported for plant cells from stoneberry, lingonberry and cloudberry (21%–36%) (Nordlund et al., 2018). Both RW and SG cells were richer in protein than previously studied berry cell lines (14%–19%) (Nordlund et al., 2018). SG cells had a relevant amount of vitamin C, meaning that the consumption of less than 30 g of SG cells would provide the daily nutritional requirement of vitamin C (80 mg/day for adults). RW cells accumulated only about one-third of the vitamin C amount, i.e. 126 mg/100 mg, but this is significantly more than previously reported 3.4 mg/100 mg (Ritala et al., 2022) and could be due to the different production systems (stirred bioreactor vs. wave bioreactor). RW cells contained vitamins D3 and E, and the consumption of 100 g of rowan cells could provide the daily nutritional requirement of vitamin D (15 µg) but not enough of vitamin E (11 mg per day). Although the presence of large amounts of vitamin D3 is not common in plants, vitamin D3 and its metabolites have been found in relatively large quantities in other species from the families Solanaceae, Poaceae and Cucurbitaceae (Göring, 2018). We hypothesize that its presence is due to the ability of RW to accumulate vitamin D3 under the culture conditions employed in their production.

According to EU regulation 1169/2011 (European Union, 2025) significant amounts of vitamins and minerals in a food product are considered relevant when 15% of the nutrient reference value is supplied by 100 g of the product. SG cells could have nutrition claims for calcium, magnesium, potassium and zinc as 100 g of this plant cell would provide 35%, 50%, 71% and 76% of the nutrient reference value, respectively. The amounts of iron, manganese and phosphorus are even higher than the reference values (2.7, 5.6 and 2-times, respectively). In the case of molybdenum, the amount in SG cells (0.84 mg/100 g) exceeded the tolerable upper intake (0.6 mg). It is noteworthy that these plant cells are presumed not to be consumed ‘as is’ but rather as an ingredient in a food product. Generally, the availability of individual ions could be limited or increased in the culture medium so that the cells accumulate less or more specific elements.

Allergen analysis is an important part of the novel food assessment to provide information to the food industry for implementing and monitoring allergen risk assessment and management processes (Tuzimski and Petruczynik, 2020). In our study, protein profiling revealed the presence of profilin-like proteins in both PCC samples. Specific profilins are pollen-related allergens and a major cause of cross-sensitization between pollen and plant-derived foods (Ruiz-García et al., 2011). Moreover, in both the SG and RW PCC samples, proteins related to metabolism and stress response were identified as putative allergens due to their reported allergenic potential in other species or closely related plant families. This underscores the need for further investigation, particularly functional allergy assays, to ensure consumer safety.

Results of the acute toxicity study of SG and RW PCCs showed that both PCCs: SG and RW, posed no death, behavioral changes, nor metabolic effects in mice at a dose of 2000 mg/kg, indicating that the LD_50_ exceeds 2 g/kg and does not present a risk of acute toxicity (OECD, 2002b). The 28-day oral subacute toxicity studies provide information on the adverse effects of repeated exposure towards the test substance over a period of time. It allows for the investigation of possible cumulative effects in target organs and dose-response relationships and provides a prediction for long-term safety (OECD, 2008). Methods include clinical observations, haematological, biochemical, relative organ weight and histopathological analysis. Significant deviations from normal values may signal adverse reactions such as metabolic disturbances, hormonal imbalances, or direct toxic effects on specific organs. The 28-day daily administration of SG plant cell cultures at 500 mg/kg posed no observed effects on the parameters measured in the study. At 1,000 mg/kg, SG caused an enlargement in the liver and lungs and increased the WBC and GRA counts, while at 2000 mg/kg, SG caused the enlargement of the liver and increased the GRA counts only. The increase in WBC and GRA counts indicates that SG amplifies immune response. This could be explained by the fact that the *Cochlearia* species contain high amounts of vitamin C, which is known to increase the production of WBCs (Brandrud, 2014; Van Gorkom et al., 2018). The increase in the relative sizes of the liver and lungs, on the other hand, may indicate potential toxicity in those organs. When liver toxicity occurs, serum/plasma ALT and AST levels increase due to damage to the liver cells, where they release these enzymes into the bloodstream (Kasarala and Tillmann, 2016). However, plasma biochemical analysis revealed that there were no significant changes in ALT and AST levels which indicate healthy liver function. Furthermore, visual histopathological scoring did not reveal any significant changes in the gross pathology for both the liver and lungs, and the increased relative organ weight of the livers and lungs were still within a normal healthy range as compared to other ICR-CD1 mice in other studies (Kyselova et al., 2003; Rao et al., 2020). Thus, these findings indicate that SG cell cultures likely would not impose any toxicity in the liver and lungs, and the no-observed-adverse-effect level (NOAEL) level was determined to be above 2,000 mg/kg. Nonetheless, further investigations could be done to confirm this, such as assessing molecular biomarkers, e.g., levels of enzymes involved in detoxification in the liver and lungs.

Like the results from the SG 28-day repeated exposure, the repeated exposure to RW cell cultures at 500 mg/kg did not affect any of the parameters measured. At 1,000 mg/kg and 2000 mg/kg of RW administration, most parameters measured showed no changes between the control and the treated mice groups, including haematological parameters, relative organ weight measurements and histological assessments. Changes in some plasma biochemical parameters were, however, noted, including an increased sodium and potassium level and a decrease in glucose levels. RW berries are known to contain polyphenols which offer antihyperglycemic and antidiabetic effects (Wei et al., 2016), which likely contributed to the lower glucose levels observed. The increase in sodium and potassium levels could be attributed to the extra sodium and potassium intake from RW PCC administration. Although an increase in sodium levels may indicate conditions of renal dysfunction leading to hypernatremia, other renal function markers (CREJ and UA) did not differ between mice administered RW and control mice of both genders; in fact, they were slightly lower, suggesting a protective effect on the kidneys. Interestingly, while the nutritional content of RW PCCs has lower potassium levels than SG PCCs, no significant increase in potassium levels was observed in mice supplemented with 2000 mg/kg SG in the 28-day subacute toxicity study. This could perhaps be attributed to RW offering better potassium bioavailability than SG, given that potassium bioavailability was known to vary among fruits and vegetables (Ceccanti et al., 2022). Further assessment of the bioavailability of nutrients in these PCCs could be investigated to confirm this observation. Nonetheless, considering that there were no significant differences in histology grading scores between organs of the control mice and mice supplemented with RW, it is safe to assume that RW PCCs pose no direct toxic effects to these organs and the NOAEL was established to be beyond 2,000 mg/kg.

While these findings demonstrate the safety of PCCs as a food ingredient for human consumption, there are limitations to consider. To move towards commercialization of food ingredients produced by PCC technology, the use of food-grade materials in their production including growth media with natural elicitors are required (Gubser et al., 2021). However, in the case of the current two cell lines, laboratory-grade reagents such as synthetic phytohormones used in the growth media have not been approved or tested for food or nutritional use, and their metabolic fate can be complex (Häkkinen et al., 2020). Therefore, the influence of growth media to PCC quality and safety is indeed an important parameter to be studied in future, especially in cases wherein new compounds are introduced into the growth media.

## 5 Conclusion

This work serves as a preliminary study characterizing the macro- and micronutrients of PCCs of SG and RW and evaluating the acute and subacute safety and toxicity of the PCCs for human consumption. The acute toxicity study results confirmed that neither mortality nor toxic were effects observed in mice treated with the two PCCs at 2000 mg/kg, indicating minimal risk of acute toxicity. Similarly, the subacute toxicity study demonstrated no mortality following 28 days of repeated oral exposure to both SG and RW at a dose of 2000 mg/kg. Considering the bioactive effects of SG, despite a slight increase in liver and lung weight, organ histopathological assessments all came out normal, and no adverse effects were observed in the body weight, haematological, and biochemical parameters. Hence, the NOAEL was determined to be >2000 mg/kg. Similarly, for the RW subacute toxicity study, an increase in sodium and potassium levels were observed in the 1,000 mg/kg mice, which indicates possible kidney and/or heart dysfunction. Despite that, all haematological, body weight, organ weight and histopathology were insignificantly different from the healthy control mice. Additionally, our findings show that RW retained its polyphenol-derived antihyperglycemic properties. Therefore, the NOAEL for RW PCCs was also determined to be >2000 mg/kg. This study offers a preliminary finding that these PCCs are safe and non-toxic for consumption as food.

## Data Availability

The datasets presented in this study can be found in online repositories. The names of the repository/repositories and accession number(s) can be found in the article/Supplementary Material.
