# Supplementary material for: Toxicological and nutritional evaluation of plant cell cultures from scurvy grass (Cochlearia danica) and rowan (Sorbus aucuparia)
Source: Front Toxicol. 2025 Oct 20;7:1655489. doi: 10.3389/ftox.2025.1655489 (PMC12580138; doi:10.3389/ftox.2025.1655489)
Supplement: Supplementary file 1 [file Supplementaryfile1.pdf]

Supplementary Materials and Methods

Supplementary Table 1. Composition of the Murashige & Skoog medium used for the growth of plant cell cultures.

Ingredients	Amount
**Carbon source**	**g/L**
Sucrose	30
**Micro Elements**	**mg/l**
CoCl2.6H2O	0.025
CuSO4.5H2O	0.025
FeNaEDTA	36.70
H3BO3	6.20
KI	0.83
MnSO4.H2O	16.90
Na2MoO4.2H2O	0.25
ZnSO4.7H2O	8.60
**Macro Elements**	**mg/l**
CaCl2	332.02
KH2PO4	170.00
KNO3	1900.00
MgSO4	180.54
NH4NO3	1650.00
**Vitamins**	**mg/l**
Glycine	2.00*
myo-Inositol	100.00
Nicotinic acid	0.50*
Pyridoxine HCl	0.50*
Thiamine HCl	0.10* or 1**
**Phytohormones**	**mg/L**
Kinetin	0.1*
Naphtalene acetic acid (NAA)	1*
2,4-Dichlorophenoxyacetic acid	0.2**

*only added for the culture of rowan PCCs

**only added for the culture of scurvy grass PCCs

Supplementary Table 2. Histopathology grading parameters of tissue specimens.

Brain								
**Parameters**	**Score**							
	0	1	2	3	4	5	6	7
**Necrosis (area)**	None	≤10%	~20%	~30%	~45%	~60%	~75%	90-100%
**Haemorrhage**	Absent	Mild	Moderate	Severe				
**Heart**								
**Parameters**	**Score**							
	0	1	2	3	4	5	6	7
**Necrosis (area)**	None	≤10%	~20%	~30%	~45%	~60%	~75%	90-100%
**Lesions**	None	Focal lesions at apex and middle of ventricles	Focal lesions over both ventricles	Confluent lesions at apex, middle of both of ventricles				
**Haemorrhage**	Minimal	None	Mild & moderate	Diffuse				
**Lungs**								
**Parameters**	**Score**							
	**0**	**1**	**2**	**3**				

**Vascular features**	**Obstruction**	None	Mild RBC obstruction	Mild RBC and vascular obstruction	Moderate RBC and vascular obstruction
	**Haemorrhage**		Blood leaking into interstitium	Areas of mild and moderate haemorrhage	Diffuse haemorrhage
**Extravascular and alveolar features**	**Inflammatory exudate**	Minimal	Mild inflammatory exudate; areas of patchy oedema with some disordered structure	Moderate	Moderate-severe
	**Alveolar thickening**			~25-50%	>50%, loss of structure with amorphous material
	**Alveolar obstruction**	Minimal	Mild	Moderate	Moderate-severe
**Bronchiole features**		Mild infiltration of inflammatory cells	Moderate infiltration of inflammatory cells, detachment of lining in some bronchioles	Complete loss of structure, detachment of lining, cellular debris, inflammatory cell exudate	

Liver

**Parameters**	**Score**				
	**0**	**1**	**2**	**3**	**4**
**Steatosis**	<5%	5-33%	33-66%	>66%	
**Fibrosis**	None	Perisinusoidal / periportal	Perisinusoidal & portal/periportal	Bridging fibrosis	Cirrhosis
**Lobular inflammation (/200x field)**	None	<2 foci	2-4 foci	>4 foci	
**Ballooning**	None	Few	Many/prominent		

Kidneys

**Parameters**	**score**			
	**0**	**1**	**2**	**3**
**Glomerular cell proliferation (of glomeruli)**	None	<25%	25-50%	>50%
**Leukocyte exudation (polymorphonuclear leukocyte per glomerulus)**	0-2	Mild	Moderate	Extensive
**Cellular crescents**	None	<25%	25-50%	>50%
**Hyaline deposits**	None	Few	Moderate	Extensive
**Glomerular sclerosis**	None	Mild	Moderate	Extensive
**Tubular atrophy**	None	Mild	Moderate	Extensive

Spleen & Thymus

**Parameters**	**Score**			
	**0**	**1**	**2**	**3**
**Lymphoid depletion**	0-10%	10-30%	30-70%	>70%

Ovaries

**Parameters**	**Score**		
	**0**	**1**	**2**
**Haemorrhage**	Normal	Moderate	Severe
**Congestion**	Normal	Moderate	Severe
**Folicular degeneration**	Normal	Moderate	Severe
**Inflammation**	Normal	Moderate	Severe

Testes

**Score**	**Criteria**
**0**	Complete spermatogenesis with many spermatozoa. Germinal epithelium of regular thickness leaving an open lumen.
**1**	Many spermatozoa but germinal epithelium disorganized with marked sloughing or obliteration of lumen.
**2**	Only a few spermatozoa are present in the section.
**3**	No spermatozoa but many spermatids present.
**4**	No spermatozoa, only a few spermatids are present.
**5**	No spermatozoa, spermatids but several or many spermatocytes present.
**6**	Only few spermatocytes and no spermatids or spermatozoa are present.
**7**	Spermatogonia are the only germ cells present.
**8**	No germ cells but Sertoli cells present.
**9**	No cells in the tubular section

Supplementary Results

Supplementary Table 3. Probable allergens identified in plant cell cultures Scurvy Grass (*Cochlearia danica*). The Protein abundance has been reported as sum of unique peptide reporter ion intensities.

Accession UniProt	Protein	Organism	Coverage [96	Abundance	Reference
Q42418	Profilin-2 (AtPROF2) (AthPRF2)	Arabidopsis thal iana (Mouse-ear cress)	36	1577.8	*89% similarity with Q42449,*
Q9FUB8	Profilin	Brassica napus (Rape)	24	216	*75% similarity with Q42449,*
Q38905	Profilin-5 (AtPROF4) (AthPRF4)	Arabidopsis thal iana (Mouse-ear cress)	14	87	*75% similarity with Q42449,*
P22953	Heat shock 70 kDa protein 1	Arabidopsis thal iana (Mouse-ear cress)	48	12213.8	*Reviewed in Shevchenko, Marina et al. Journal of asthma and allergy vol. 13 757-772.5 Jan. 2021, doi:10.2147/JAAS288886*
Q9LKR3	Heat shock 70 kDa protein BIPI	Arabidopsis thal iana (Mouse-ear cress)	42	18977.1	
P22954	Heat shock 70 kDa protein 2	Arabidopsis thal iana (Mouse-ear cress)	41	1618.3	
O65719	Heat shock 70 kDa protein 3	Arabidopsis thal iana (Mouse-ear cress)	40	1393.1	
Q9LHA8	Heat shock 70 kDa protein 4	Arabidopsis thal iana (Mouse-ear cress)	34	224.1	
Q9LTX9	Heat shock 70 kDa protein 7	Arabidopsis thal iana (Mouse-ear cress)	24	1892.1	
Q9LDZO	Heat shock 70 kDa protein 10	Arabidopsis thal iana (Mouse-ear cress)	23	6071.8	
Q8HIB3	Heat shock 70 kDa protein BIP3	Arabidopsis thal iana (Mouse-ear cress)	10	406.3	
Q9S9N1	Heat shock 70 kDa protein 5	Arabidopsis thal iana (Mouse-ear cress)	19	571.7	
Q8GUM2	Heat shock 70 kDa protein 9	Arabidopsis thal iana (Mouse-ear cress)	16	1425.2	
Q9S7CO	Heat shock 70 kDa protein 14	Arabidopsis thal iana (Mouse-ear cress)	13	4638.3	
F4JMJ1	Heat shock 70 kDa protein 17	Arabidopsis thal iana (Mouse-ear cress)	10	4530.6	
P93819	Malate dehydrogenase 1	Arabidopsis thal iana (Mouse-ear cress)	28	992.1	*Pastor, Coriosetaf. International archives of allergy and immunology vol. 149,4 (2009):291-8. doi:10.1159/000205574*
Q9SN86	Malate dehydrogenase	Arabidopsis thal iana (Mouse-ear cress)	16	2938	
Q9ZP06	Mal ate dehydrogenase 1	Arabidopsis thal iana (Mouse-ear cress)	16	3309.5	
O82399	Mal ate dehydrogenase 1	Arabidopsis thal iana (Mouse-ear cress)	16	192.1	
Q9SKP6	Triosephosphate isomerase	Arabidopsis thal iana (Mouse-ear cress)	31	2009.6	*Pastor, Coriosetaf. International archives of allergy and immunology vol. 149,4 (2009):291-8. doi:10.1159/000205574; Yang, Yang et al. Molecular immunology vol. 85 (2017): 35-46. doi:10.1016/j.molimm.2017.02.004*
P48491	Triosephosphate isom erase	Arabidopsis thal iana (Mouse-ear cress)	29	8963.3	
O65570	V.Ilin4	Arabidopsis thal iana (Mouse-ear cress)	11	2046.3	*Mittermann, Irene et al. FEBS letters vol. 579,17 (2005):3807-13. doi:10.1016/j.febslet.2005.05.066*
O81644	V.Ilin.2	Arabidopsis thal iana (Mouse-ear cress)	18	3650.2	
Q9ZU52	Fructose-bisphosphate aldolase 3	Arabidopsis thaliana (Mouse-ear cress)	22	2181.1	*Ventura, Anne K R et al. The World Allergy Organization journal vol. 16,12 100845.22 Nov. 2023, doi:10.1016/j.woojou.2023.100845*
Q9SR37	Beta-glucosidase 23 (AtBGLU23)	Arabidopsis thaliana (Mouse-ear cress)	15	1442.4	*Ogino, Ryohei et al. Allergology International official journal of the Japanese Society of Allergology vol. 70,2 (2021):215-222. doi:10.1016/j.alit.2020.09.005*
Q9FGY1	Beta-D-xylosidase 1 (AtBXL1)	Arabi dopsisthaliana (Mouse-ear cress)	11	119.4	*Sander, I et al. The journal of allergy and clinical immunology vol. 102,2 (1998):256-64. doi:10.1016/s0091-6749(98)70109-5*
P04796	Glyceraldehyde-3-phosphate dehydrogenase	Sinapis alba (White mustard) (Brassica hirta)	48	587.7	*Annals of allergy, asthma & immunology: official publication of the American College of Allergy, Asthma, & Immunology vol. 106,6 (2011):545-7. doi:10,1016/j.anai.2011.03.008*
PODH95	Calmodulin-l (CaM-I)	Arabidopsis thaliana (Mouse-ear cress)	54	6696.3	*Gómez-Esquivel, Mónica Luz et al. Molecular immunology vol. 132 (2021):150-156, doi:10.1016/j.molimm.2021.01.031*
Q96528	Catalase-I (EC 1.11.1.6)	Arabi dopsis thal iana (Mouse-ear cress)	17	624.4	*Nikolić, Jasna et al. Journal of proteomics vol. 175 (2018):87-94. doi:10.0016/j.jprot.2018.01.007*
Q42592	L-ascorbate peroxidase S	Arabi dopsis thaliana (Mouse-ear cress)	15	385.4	*Experimental allergy journal of the British Societyfor Allergy and Clinical Immunology vol. 34,11*

Supplementary table 4. Putative and probable allergens identified in plant cell cultures Rowan (*Sorbus aucuparia*). The protein abundance has been reported as sum of unique peptide reporter ion intensities.

Accession UniProt	Protein	Organism	Coverage [%]	Abundance	Reference
Q8GSL5	Profilin	Pyrus communis	28	283.9	
Q9XF38	Profilin	Prunus armeniaca	23	2420	UniProt
Q8GT39	Profilin	Prunus persica	18	1438.5	
Q38905	Major strawberry allergen Fra a 1.06	Fragaria ananassa	54	2629.7	
P22953	Major strawberry allergen Fra a 1-3	Fragaria ananassa	52	11020.8	UniProt
Q9LKR3	Major strawberry allergen Fra a 1.07	Fragaria ananassa	51	2883.4	
Q38906	Major strawberry allergen Fra a 1.06	Fragaria ananassa	49.33	5765	
O50001	Major allergen Pru ar 1	Prunus armeniaca	15	2026.6	UniProt
O81355	Phenylcoumaran benzylic ether reductase Pyrc5	Pyrus communis	10	2286.9	UniProt
P48976	Calmodulin	Malus domestica	50	2288	*Gómez-Esquivel, Mónica Luz et al. Molecular immunology vol. 132 (2021): 150-156. doi:10.1016/j.molimm.2021.01.031*
Q9M4S8	Triosephosphate isomerase, chloroplastic	Fragaria ananassa	31	1809.5	*Yang, Yang et al. Molecular immunology vol. 85 (2017): 35-46. doi:10.1016/j.molimm.2017.02.004*
P83373	Malate dehydrogenase, mitochondrial	Fragaria ananassa	13	2439.9	*Pastor, Carlos et al. International archives of allergy and immunology vol. 149,4 (2009): 291-8. doi:10.1159/000205574*

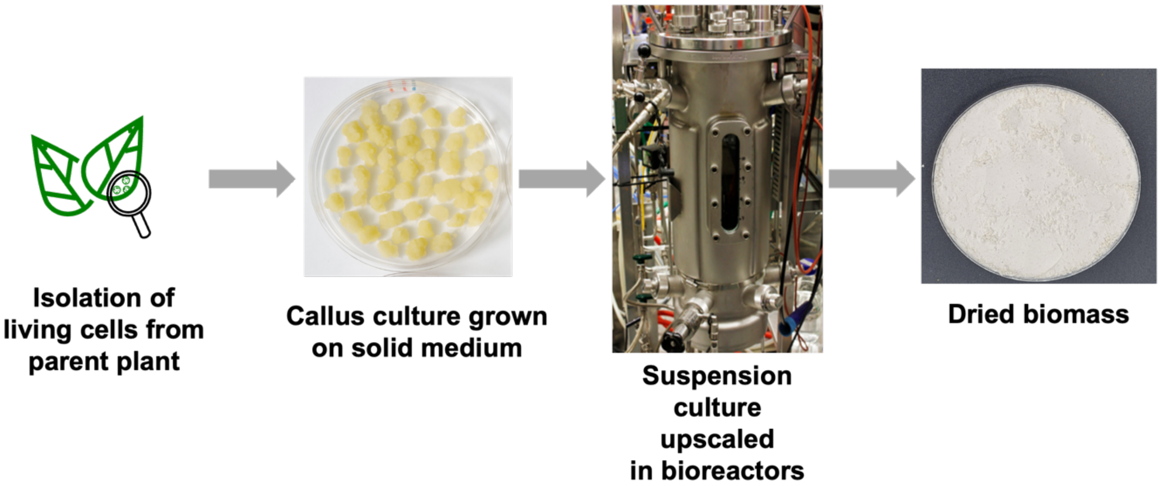

Supplementary Figure 1. Workflow of Plant Cell Culture Production from Parent Plant to Dried Biomass

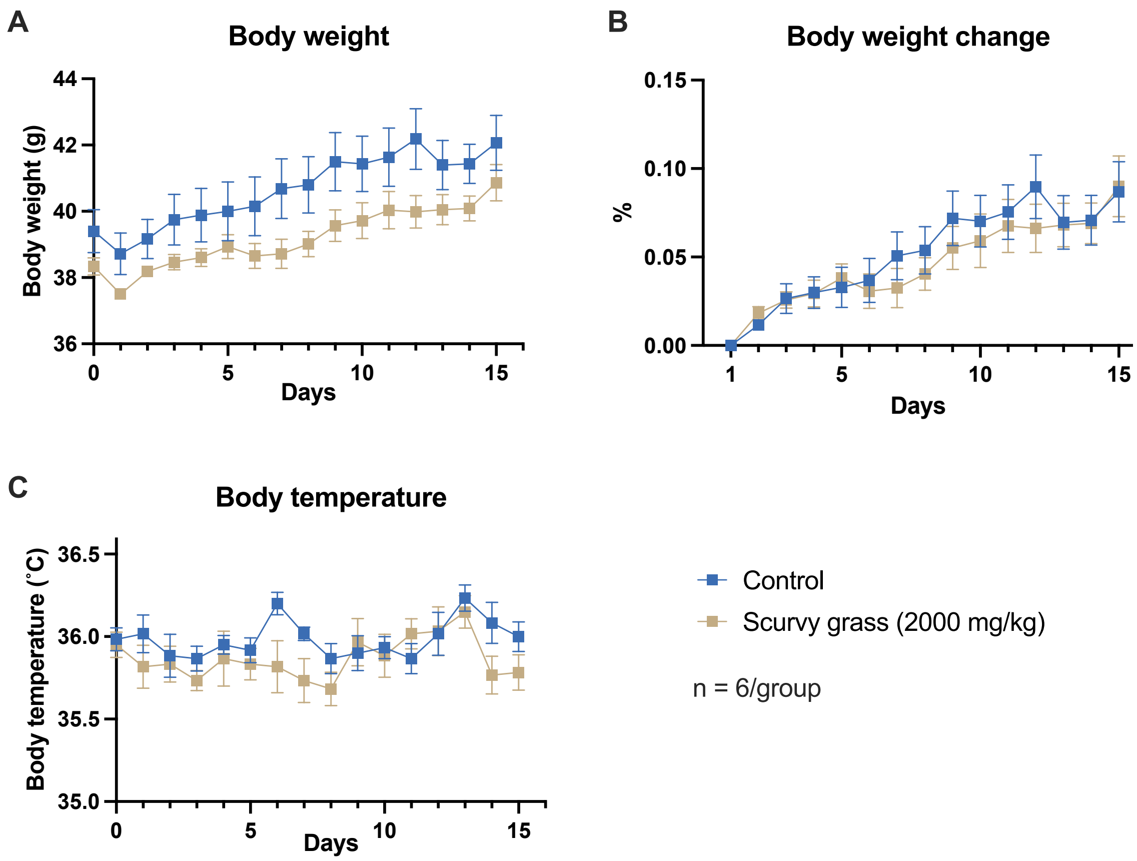

Supplementary Figure 2. Results of the scurvy grass 14-day acute toxicity study. (**A)** Body weight of mice. (**B)** Relative change in body weight from the start of study. (**C)** Body temperature of mice. No statistical differences were noted between the two groups.

Supplementary Table 5. Haematological parameters of male ICR-CD1 mice in the 14-days acute toxicity study of Scurvy Grass (SG). Values are expressed as mean ± SD

	Control (n = 6)	SG (2000 mg/kg) (n = 6)
WBCs (10^9 g/L)	2.14 ± 0.56	2.71 ± 0.86
LYM (10^9 g/L)	1.12 ± 0.34	1.17 ± 0.31
MID (10^9 g/L)	0.16 ± 0.08	0.14 ± 0.06
GRA (10^9 g/L)	0.86 ± 0.22	1.40 ± 0.63
RBCs (10^{12} g/L)	7.60 ± 0.28	7.40 ± 0.78
HGB (g/L)	127.17 ± 6.68	127.25 ± 12.51
HCT (%)	36.40 ± 11.85	39.97 ± 3.48
MCH (pg)	16.73 ± 0.36	17.24 ± 0.56
MCHC (gL)	307.83 ± 8.93	318.42 ± 14.57*
MCV (fL)	54.35 ± 1.66	54.14 ± 2.09
Platelets (10^9 g/L)	868.83 ± 47.66	863.67 ± 145.89

*$p < 0.05$ Control vs SG

Supplementary Table 6. Organ-body weight ratio for male ICR-CD1 mice in the acute toxicity study of Scurvy Grass (SG). Values are expressed as mean ± SD

	**Control** (n = 6)	**SG (2000 mg/kg)** (n = 6)
Brain	1.13 ± 0.11	1.15 ± 0.12
Heart	0.44 ± 0.03	0.49 ± 0.05
Lungs	0.52 ± 0.06	0.59 ± 0.04*
Liver	6.04 ± 0.19	6.29 ± 0.35
Kidneys	1.60 ± 0.14	1.55 ± 0.11
Spleen	0.31 ± 0.04	0.34 ± 0.08
Thymus	0.10 ± 0.03	0.09 ± 0.02
Testes	0.67 ± 0.12	0.71 ± 0.06

*$p < 0.05$ Control vs SG

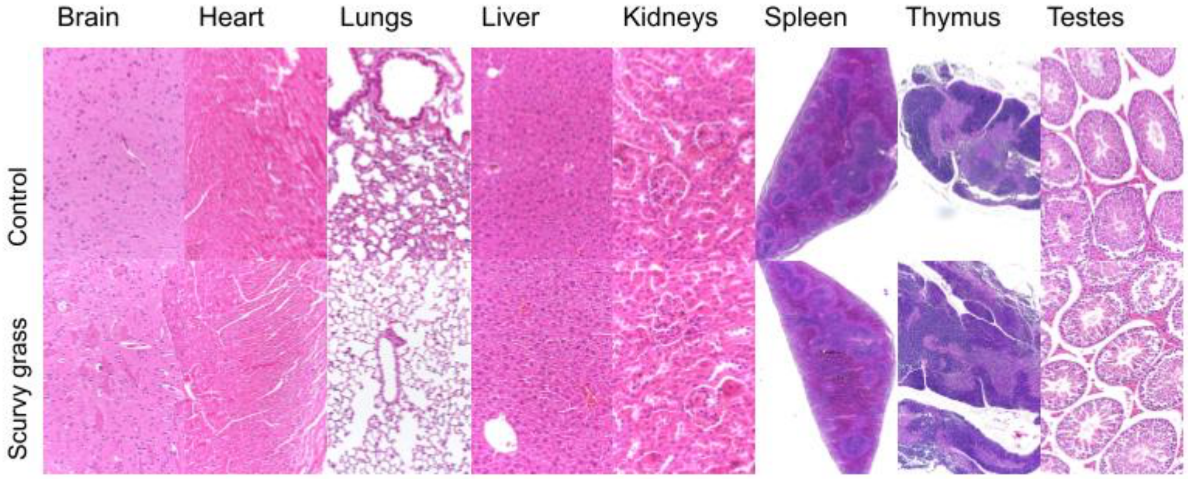

Supplementary Figure 3. H&E-stained sections of brain (2000X), heart (2000X), lungs (200X), liver (2000X), kidneys (2000X), spleen (400X), thymus (400X) and testes (2000X) of control and SG-treated mice in the 14-day acute oral toxicity study at 2000 mg/kg.

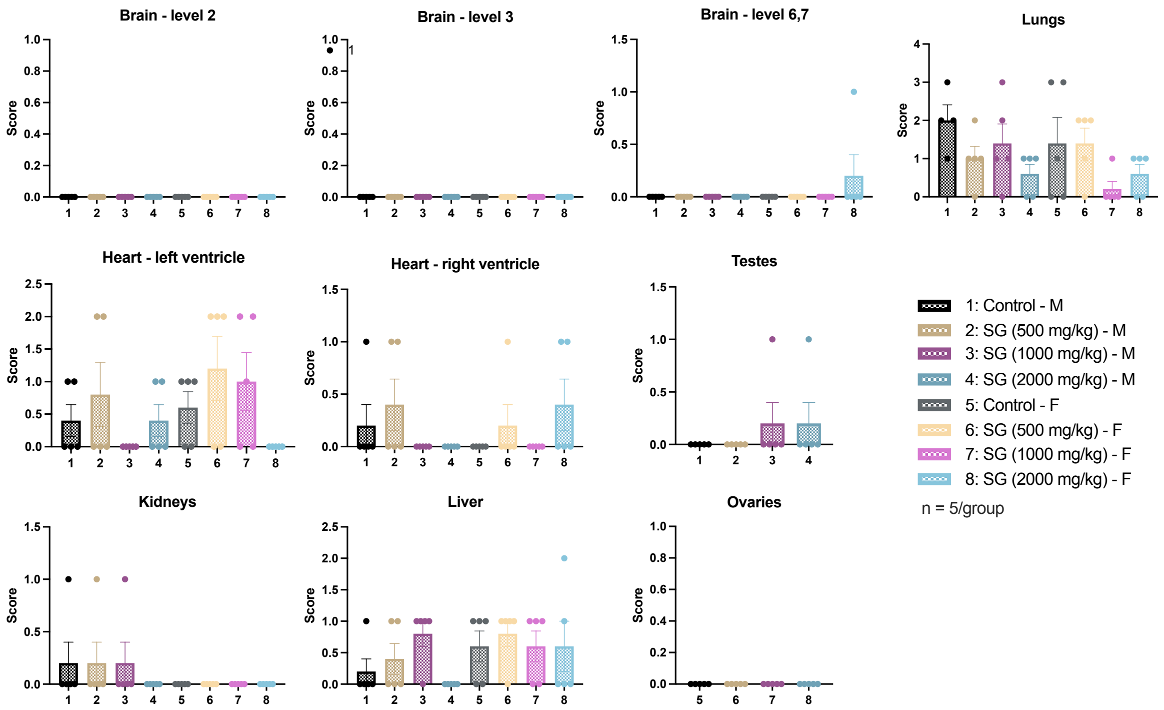

Supplementary Figure 4. Histopathological analysis scoring of organs and tissues for the 28-day subacute oral toxicity study of Scurvy Grass (SG) cell cultures.

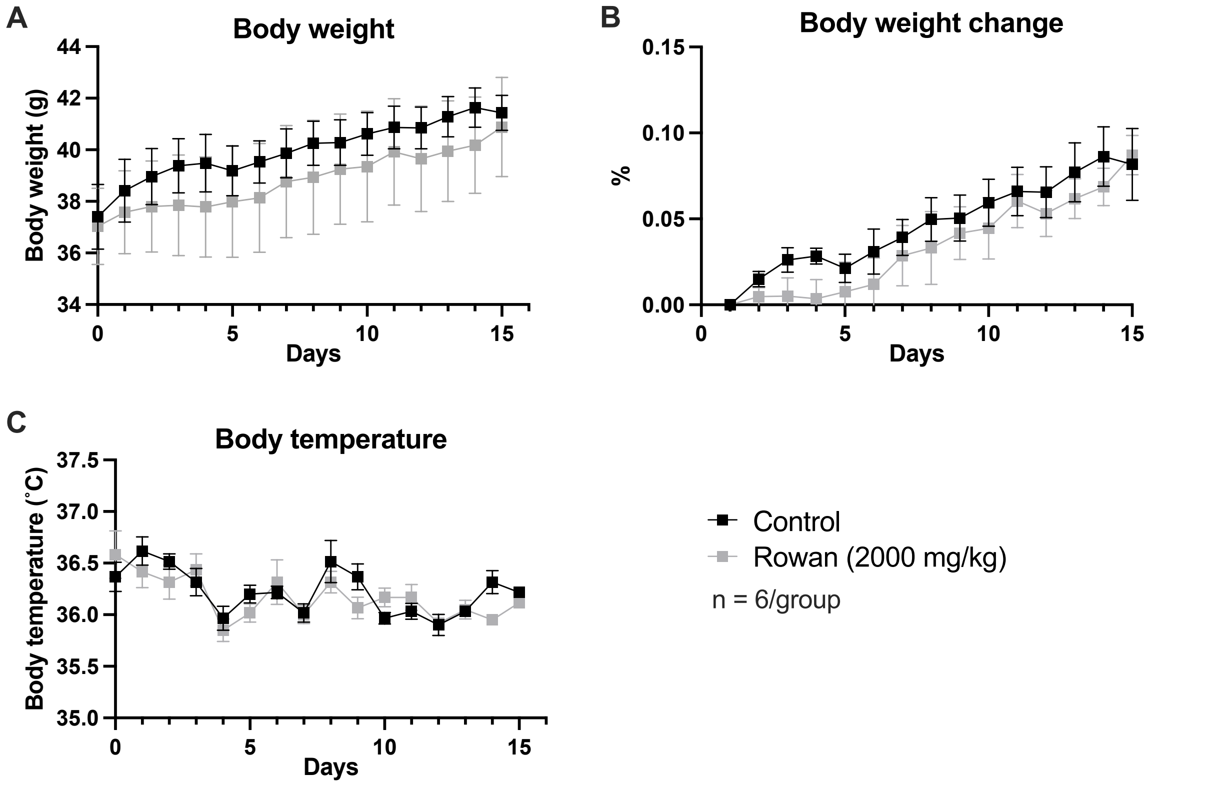

Supplementary Figure 5. Results of the rowanberry 14-day acute toxicity study. (**A**) Body weight of mice. (**B**) Relative change in body weight from the start of study. (**C**) Body temperature of mice. No statistical differences were noted between the two groups.

Supplementary Table 7. Organ-body weight ratio for male ICR-CD1 mice in the acute toxicity study of Rowan (RW). Values are expressed as mean ± SD

	**Control** (n = 6)	**RW (2000 mg/kg)** (n = 6)
Brain	1.09 ± 0.19	1.12 ± 0.14
Heart	0.44 ± 0.01	0.47 ± 0.05
Lungs	0.64 ± 0.04	0.68 ± 0.08
Liver	5.99 ± 0.22	5.03 ± 2.48
Kidneys	1.63 ± 0.07	1.61 ± 0.12
Spleen	0.27 ± 0.04	0.38 ± 0.17
Thymus	0.14 ± 0.03	0.12 ± 0.04
Testes	0.66 ± 0.04	0.74 ± 0.10

No statistical significance between the control group and treatment group was observed

Supplementary Table 8. Haematological parameters of male ICR-CD1 mice in the 14-days acute toxicity study of Rowan (RW). Values are expressed as mean ± SD

	**Control (n = 6)**	**RW (2000 mg/kg) (n = 6)**
WBCs (10^9 g/L)	2.63 ± 0.94	3.03 ± 0.71
LYM (10^9 g/L)	1.40 ± 0.60	1.20 ± 0.40
MID (10^9 g/L)	0.18 ± 0.09	0.18 ± 0.06
GRA (10^9 g/L)	1.06 ± 0.44	1.65 ± 0.53
RBCs (10^{12} g/L)	7.59 ± 0.28	7.69 ± 0.26
HGB (g/L)	128.50 ± 5.39	127.50 ± 5.13
HCT (%)	301.17 ± 4.45	305.50 ± 4.55
MCH (pg)	16.95 ± 0.23	16.62 ± 0.57
MCHC (gL)	56.20 ± 0.93	54.43 ± 2.15
MCV (fL)	42.67 ± 1.77	41.82 ± 1.64
Platelets (10^9 g/L)	802.33 ± 44.57	765.50 ± 87.24

No statistical significance between the control group and treatment group was observed

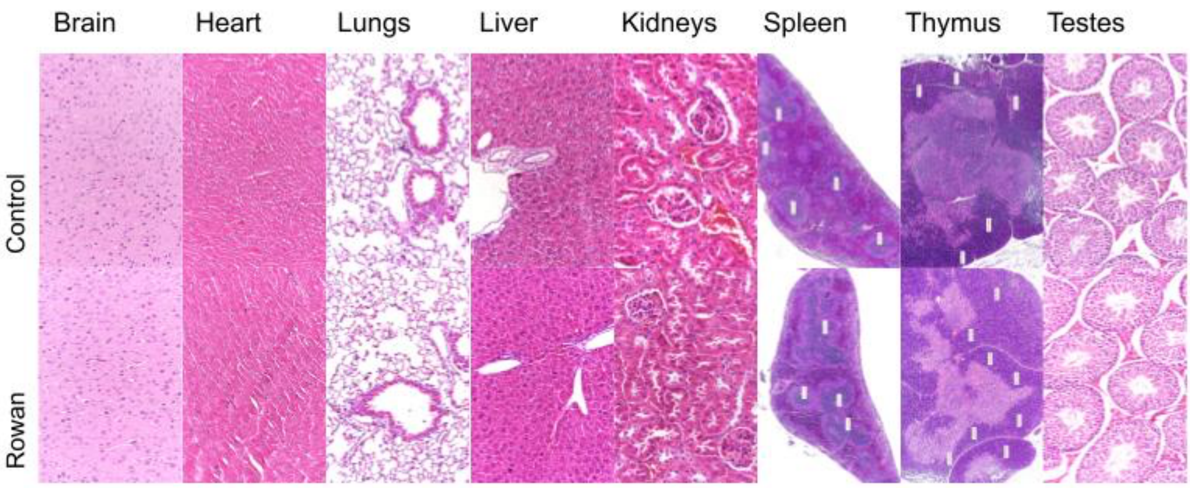

Supplementary Figure 6. Haematoxylin and eosin-stained sections of brain (200X), heart (200X), lungs (200X), liver (200X), kidneys (200X), spleen (40X), thymus (40X) and testes (200X) of control and Rowan-treated mice in 14-day acute oral toxicity study.

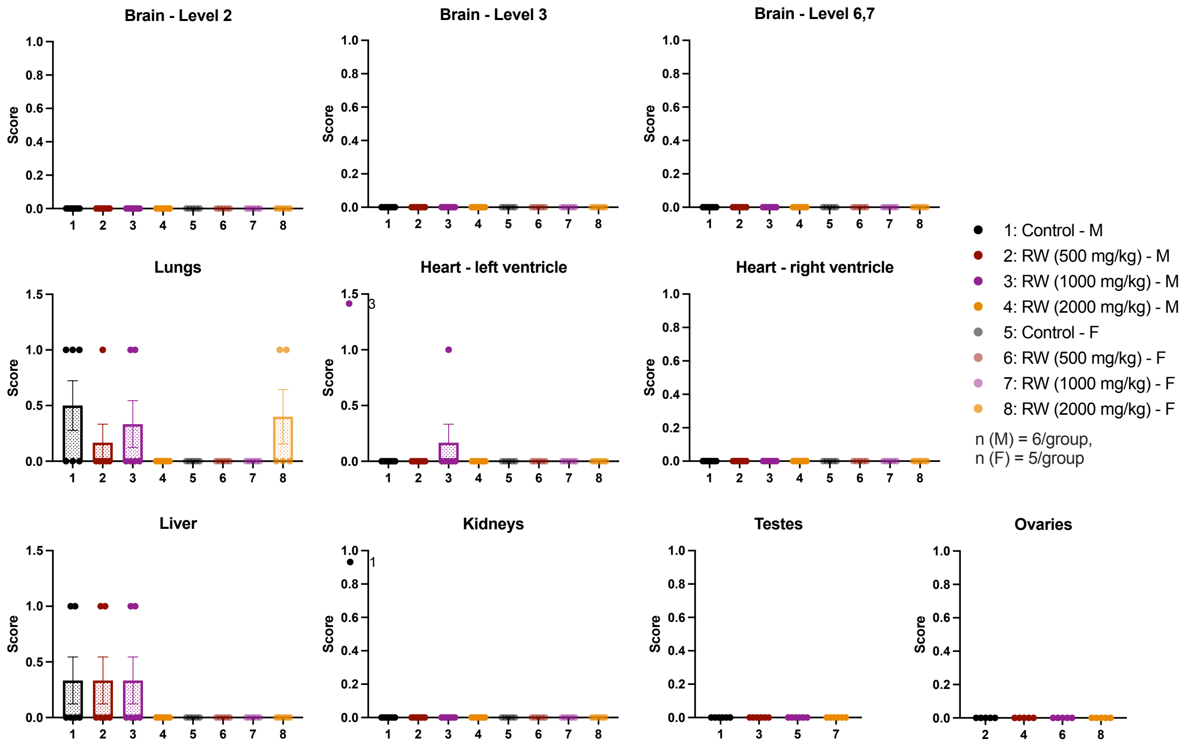

Supplementary Figure 7. Histopathological analysis scoring of organs and tissues for the 28-day subacute oral toxicity study of rowan (RW) cell cultures.
